# Exploring Attitudes Toward AI-Based Contactless Sensors in Health Among Five Stakeholder Groups: Qualitative Study

**DOI:** 10.2196/75783

**Published:** 2026-04-24

**Authors:** Eva Maria Hille, Hannah Bleher, Birte Coppers, Sophie Engelhardt, Bjoern Eskofier, Lorenz Garbe, Maria Heckel, Anna-Maria Liphardt, Alina Manlig, Nicolas Rohleder, David Samhammer, Carla Spielmann, Matthias Braun

**Affiliations:** 1 Department of (Social) Ethics, University of Bonn, Am Hofgarten 8, Bonn, 53113, Germany; 2 Department of Medicine 3 - Rheumatology and Immunology, Friedrich-Alexander-Universität Erlangen-Nürnberg and Uniklinikum Erlangen, Erlangen, Germany; 3 Deutsches Zentrum für Immuntherapie (DZI), Friedrich-Alexander-Universität Erlangen-Nürnberg and Uniklinikum Erlangen, Erlangen, Germany; 4 Didaktik der sprachlichen und ästhetischen Fächer (EW 4), Faculty of Education, Universität Hamburg, Hamburg, Germany; 5 Department Artificial Intelligence in Biomedical Engineering, Friedrich-Alexander-Universität Erlangen-Nürnberg, Erlangen, Germany; 6 Institute of AI for Health, Translational Digital Health Group, Helmholtz Zentrum München, München, Germany; 7 Chair of AI-supported Therapy Decisions, Ludwig-Maximilians-Universität München, Munich, Bavaria, Germany; 8 Department of Palliative Medicine, Universitätsklinikum Erlangen, Friedrich-Alexander-Universität Erlangen-Nürnberg, Erlangen, Germany; 9 Comprehensive Cancer Center Erlangen-EMN (CCC ER-EMN), Erlangen, Germany; 10 Chair of Health Psychology, Friedrich-Alexander-Universität Erlangen-Nürnberg, Erlangen, Germany; 11 Social Science Institute of the EKD, Hannover, Germany; 12 Institute of Sociology, Friedrich-Alexander-Universität Erlangen-Nürnberg, Erlangen, Germany

**Keywords:** attitudes, values, contactless sensors, health, artificial intelligence

## Abstract

**Background:**

The rapid rise of artificial intelligence–based contactless sensors (AI-CS) is expected to significantly transform how patients are measured, monitored, and understood through a versatile, noninvasive approach to data collection and health assessment. However, there is a lack of empirical research specifically focusing on AI-CS in health. Moreover, existing studies tend to focus on medical or patient perspectives, while neglecting other stakeholders such as researchers, political actors, or the general public.

**Objective:**

The study aims to provide an in-depth empirical ethical analysis and, through a multistakeholder approach, a uniquely comprehensive overview by addressing the research question: what are the attitudes of different stakeholders (patients, health care professionals, researchers, political stakeholders, and the general public) toward AI-CS and their applications in health?

**Methods:**

We conducted a cross-sectional study with 104 participants using a semistructured interview guide. Interviews were analyzed using qualitative content analysis with ATLAS.ti software (ATLAS.ti Scientific Software Development GmbH), following a 3-component model of feelings, thoughts, and behavioral aspects.

**Results:**

The results of the study provide an in-depth analysis of attitudes toward AI-CS in health among different stakeholders. Overall, the results show a high level of openness to AI-CS in health across all stakeholder groups. In terms of feelings and their correlation with behavioral aspects, 2 key trends emerged: first, greater experience and knowledge correlated with a reduced tendency to react emotionally. Second, participants with positive experiences with technologies were generally more open and positive toward contactless sensors. The combined findings on thoughts and behavioral aspects highlighted 3 key tensions—around contact(lessness) and the importance and ambivalence of touch, between protection and surveillance (particularly regarding path- and context-dependency) and between the benefits and challenges of unobtrusiveness (especially in relation to control and governance implications). In addition, the analysis revealed the need for information and consent about AI-CS and clarified possible technical implementations and fields of application.

**Conclusions:**

This study provides a comprehensive and empirically grounded ethical analysis of stakeholder attitudes toward AI-CS in health. The findings offer valuable guidance for the responsible development, implementation, and governance of AI-CS in health care contexts.

## Introduction

The emergence of new artificial intelligence (AI)-based technologies has opened up new possibilities in health. Among these, sensor technologies have emerged as a versatile tool for data collection and health monitoring. Although still in their infancy, AI-based contactless sensors (AI-CS) are partly used in telemedicine, eHealth, and home-based medical apps and are thus proposed to change protocols and procedures in health. In this study, we aim to better understand and analyze the attitudes of different stakeholders toward the use of AI-CS in health: patients, health care professionals (HCPs), political stakeholders, researchers, and the general public. Of particular interest are the 2 important characteristics of AI-CS (their contactless and unobtrusive character) and how they are perceived in health data collection.

A better understanding of the attitudes of the involved stakeholders is crucial, as various studies have shown that the mere possibility of new technical systems also brings about changes in moral values and sociotechnical practices [1,2]. We decided on the term attitudes, considering it as an umbrella term and referring to the understanding that “[a]ttitudes are a complex combination of things we tend to call personality, beliefs, values, behaviors, and motivations” [3]. We do not limit attitudes to a temporal aspect by dividing them into states and traits. Instead, we follow the distinction “[…] between the inner tendency that is attitude and the evaluative responses that express attitudes” [4], to acknowledge the fact that a person can hold several attitudes at the same time [4,5]. Accordingly, the expression of attitudes is composed of cognitions (often referred to as thoughts or beliefs), affects (often called feelings or emotions), and behaviors (ie, actions) [4].

Several quantitative and some qualitative empirical interview studies have investigated the attitudes of certain stakeholder groups toward AI applications in health in order to predict the likelihood of their acceptance. Attitude studies have mainly focused on opportunities (primarily accuracy [6], efficiency, and enhanced workflow and speed [7]) and challenges such as the lack of human empathy [8-10], data security, and concerns about accuracy and diagnostic quality [11,12], leading to a lack of confidence in the results [13,14]. A survey in the United States found that people often see both the opportunities and challenges, while those who take sides are less optimistic about the potential benefits and more concerned about the potential risks [15,16]. People were more open-minded to the use of AI in cases where human decision-making, care, or other processes are merely enhanced by the technology, rather than replaced [16,17]. In particular, quantitative interview studies with physicians and medical students revealed an eagerness to incorporate AI into health [10,14,18].

The current state of research shows 2 intertwined deficiencies that lead to the research gap we are addressing: first, attitudes toward contactless sensor technologies in health have been less explored than those toward AI-based applications in general. Empirical research into sensor technologies in health has mostly been conducted in the field of smart homes, as a way to enable older adults to remain independent at home by monitoring their health status (eg, [19]). However, sensor technologies in health can serve a multitude of different options. In various areas of health, motion, biometric, and light sensors (radar sensors and photonic sensing cameras, including infrared cameras and depth cameras), most of which operate unobtrusively, are of great importance (eg, [20,21]). Contactless sensor technologies can be used to detect and assess the physiological states and functions of the human body (eg, the musculoskeletal systems with diseases such as inflammatory joint disease), as well as human behavior [22]. Current research reports on continuous radar-based monitoring of stress and mental health and its potential for emergency detection, as well as radar-based machine learning applications for biomedical monitoring, including gait and motion analysis, along with sleep analysis [22]. It is well established that movement is impaired as the disease progresses (eg, in the case of Parkinson disease [22] or rheumatoid arthritis [23]), while disease-specific functional models of the body still need to be explored. Machine learning methods could provide additional potential for better disease monitoring by identifying fine-tuned and disease-specific biomarkers of functional impairment [22]. Although the potential of AI-CS has been outlined, there remains a lack of empirical research, particularly with regard to attitudes.

Second, the few existing studies on attitudes toward sensor technologies, and even more so on AI in health, tend to focus on only 1 or 2 stakeholder groups, with the exception of [6,13,24], which address 3 different stakeholder groups. HCPs [24-26] and patients [6,24,27-31] were most frequently interviewed, sometimes also relatives [6,30], caregivers [24], or visitors [32]. This is a severe problem, as there is ample evidence that data collection and measurement in health and medicine are often a multifactorial and multiactor endeavor [33].

The combination of these two shortcomings, (1) the lack of empirical research on contactless sensor technologies in health and (2) the lack of a broad stakeholder perspective (including nonmedical and nonpatient perspectives, such as researchers or political stakeholders) has not yet been explored in any empirical study. To address both of these deficiencies, we have developed our research question as follows: What are the attitudes of different stakeholders (patients, HCPs, researchers, political stakeholders, and the general public) toward AI-CS and their applications in health?

## Methods

### Overview

**Figure 1.**
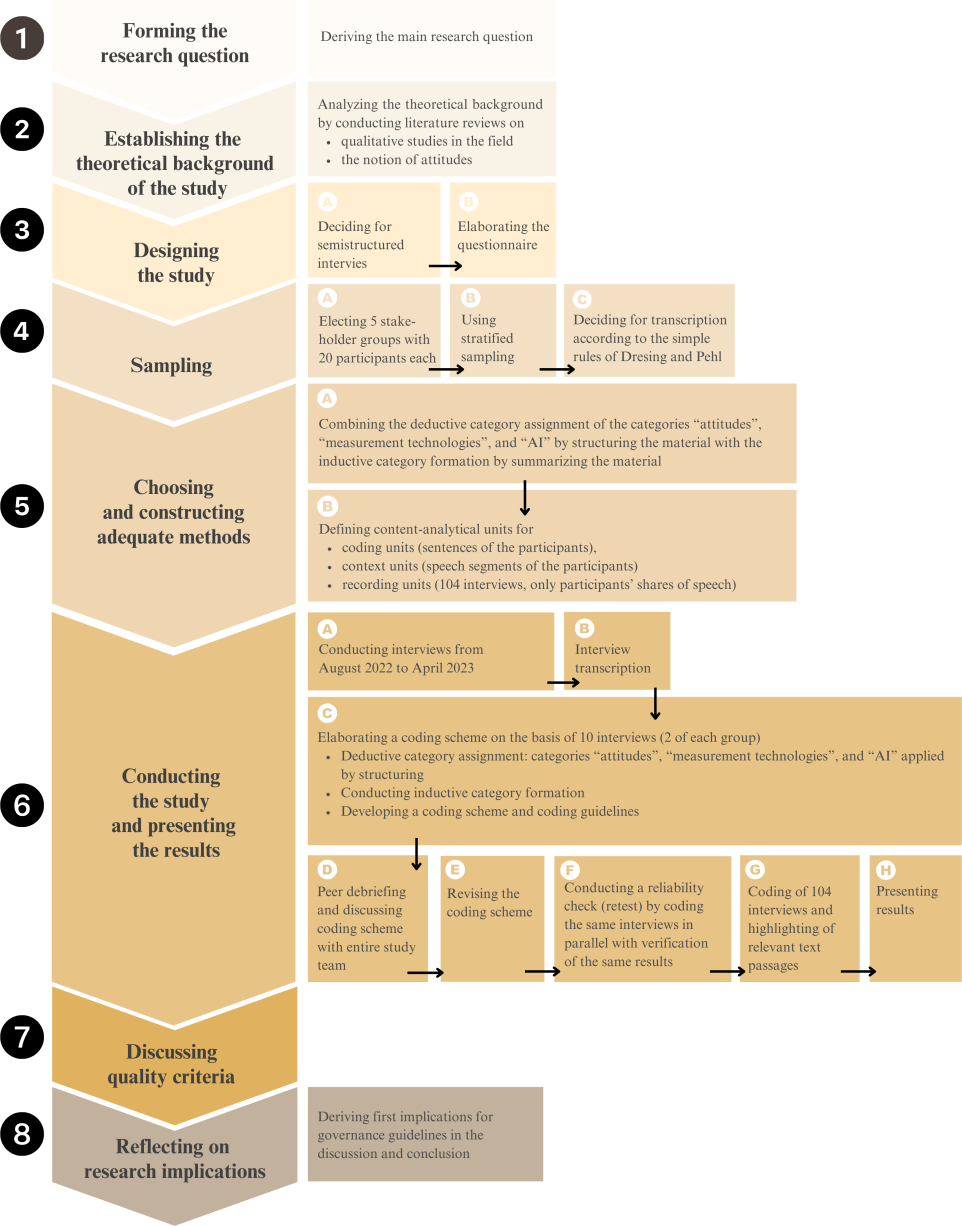
Scheme of the study design and research process (following the eight research process steps, as formulated byMayring [35]. AI: artificial intelligence.

We conducted a cross-sectional study [34] to analyze the attitudes of different stakeholder groups toward AI-CS in health. The research process (Figure 1) is based on the 8 steps of qualitative coding analysis [35]. It follows the COREQ (Consolidated Criteria for Reporting Qualitative Research) checklist [36] (Checklist 1).

### Ethical Considerations

This study was approved by the Institutional Ethics Committee of FAU Erlangen-Nürnberg on December 23, 2020 (478_20 B). Prior to data collection, pilot testing the interview guide allowed us to select AI-CS application examples to minimize anxiety induced by discussions of monitoring at the start of the interview.

Prior to the interview, participants were provided with information and reminded that they could withdraw from the interview at any time. Written informed consent was obtained from all participants. The participants did not receive financial compensation. All data were analyzed pseudonymously.

### Sampling and Recruitment

Stakeholder groups were selected through stratified sampling [37] along the idea of an innovation pipeline approach [38]. By understanding the use of AI-CS in its complexity, particularly its impact on different stakeholders and societal levels, we identified 5 relevant stakeholder groups (Figure 1, step 4) that are likely to come into contact with AI-CS, in its development, regulation, and application: (1) patients who might be monitored by using AI-CS, (2) HCPs using these sensors, (3) researchers who develop and investigate AI-CS, (4) political stakeholders who decide on their use, and (5) unaffiliated representatives of the general public. To be included, participants had to be at least 18 years old, able to complete the interview in English or German, willing to sign a consent form, and agree to be audio recorded.

The study was widely promoted nationally and internationally in medical institutions (to reach patients and HCPs), research networks (to target researchers), associations, public places, and social media (to reach political stakeholders and the general public). To ensure a similar number of interviews in each group, patients were approached personally in hospitals and other medical practices in a second phase, and political stakeholders by contacting ministries, nongovernmental organizations, and individuals. To avoid overrepresentation of people with particular sociodemographic characteristics, recruitment took place in deliberately chosen public locations (eg, hospitals and shops). We proceeded with the recruitment process, aiming to recruit at least 20 participants [39] from each stakeholder group in order to ensure that the participants represented a range of perspectives and characteristics (Table 1).

**Table 1.** Characteristics and sociodemographic status of participants.

Characteristics	All participants (n=104)	Patients (n=22)	Health care professionals (n=23)	Researchers (n=25)	Political stakeholder (n=8)	General public (n=26)
Gender, n (%)					
Female	57 (55)	11 (50)	19 (83)	11 (44)	2 (25)	14 (54)
Male	47 (45)	11 (50)	4 (17)	14 (56)	6 (75)	12 (46)
Divers	0 (0)	0 (0)	0 (0)	0 (0)	0 (0)	0 (0)
Age (y), mean (SD; range)	42 (16.6; 20‐87)	50 (18.6; 22‐87)	44 (15.2; 26‐66)	32 (8.9; 24‐60)	34 (6.7; 26‐44)	43 (19.1; 20‐76)
Highest level of education, n (%)						
No degree	1 (1)	1 (5)	0 (0)	0 (0)	0 (0)	0 (0)
Qualified secondary school leaving certificate	4 (4)	2 (9)	0 (0)	0 (0)	0 (0)	2 (8)
Technical baccalaureate	1 (1)	0 (0)	0 (0)	0 (0)	0 (0)	1 (4)
A-level equivalent	9 (9)	2 (9)	2 (9)	0 (0)	0 (0)	5 (19)
Completed apprenticeship	7 (7)	4 (18)	3 (13)	0 (0)	0 (0)	0 (0)
Technical college or technical school or trade school	4 (4)	2 (9)	1 (4)	0 (0)	0 (0)	1 (4)
Master craftsman’s diploma	1 (1)	1 (5)	0 (0)	0 (0)	0 (0)	0 (0)
Bachelor’s degree	11 (11)	1 (5)	4 (17)	0 (0)	0 (0)	6 (23)
Master’s degree	47 (45)	9 (41)	5 (22)	19 (76)	5 (63)	9 (35)
Doctorate	19 (18)	0 (0)	8 (35)	6 (24)	3 (38)	2 (8)
Marital status^a^, n (%)						
Living in partnerships	71 (68)	16 (73)	17 (74)	17 (68)	7 (88)	14 (54)
Single	30 (29)	6 (27)	5 (22)	7 (28)	1 (13)	11 (42)
Custodian of a minor	8 (8)	0 (0)	1 (4)	2 (8)	4 (50)	1 (4)
Caregiver for relatives	1 (1)	0 (0)	0 (0)	0 (0)	0 (0)	1 (4)
Not specified	1 (1)	0 (0)	1 (4)	0 (0)	0 (0)	0 (0)
Professional situation, n (%)						
Working	67 (64)	10 (45)	16 (70)	22 (88)	6 (75)	13 (50)
Apprenticeship	15 (14)	3 (14)	3 (13)	2 (8)	0 (0)	7 (27)
Pension	12 (12)	6 (27)	2 (9)	0 (0)	0 (0)	4 (15)
Started working	10 (10)	1 (5)	2 (9)	5 (20)	2 (25)	0 (0)
On sick leave	4 (4)	2 (9)	1 (4)	0 (0)	0 (0)	1 (4)
Other	3 (3)	0 (0)	0 (0)	0 (0)	0 (0)	3 (12)
Unemployed	2 (2)	0 (0)	1 (4)	0 (0)	0 (0)	1 (4)
Job change	1 (1)	0 (0)	0 (0)	1 (4)	0 (0)	0 (0)
Health insurance n (%)						
Public health insurance	83 (80)	17 (77)	19 (83)	21 (84)	6 (75)	20 (77)
Private	20 (19)	5 (23)	4 (17)	4 (16)	2 (25)	5 (19)
Other	1 (1)	0 (0)	0 (0)	0 (0)	0 (0)	1 (4)
Monthly expenses for technical apps and devices (€)^b^, n (%)						
No money	10 (10)	4 (18)	4 (17)	1 (4)	0 (0)	1 (4)
1‐10	17 (16)	4 (18)	2 (9)	4 (16)	1 (13)	6 (23)
11‐25	25 (24)	4 (18)	9 (39)	6 (24)	2 (25)	4 (15)
26‐50	27 (26)	6 (27)	4 (17)	8 (32)	1 (13)	8 (31)
51‐100	20 (19)	4 (18)	2 (9)	5 (20)	3 (38)	6 (23)
101‐250	1 (1)	0 (0)	0 (0)	1 (4)	0 (0)	0 (0)
>250	1 (1)	0 (0)	0 (0)	0 (0)	1 (13)	0 (0)
Not specified	3 (3)	0 (0)	2 (9)	0 (0)	0 (0)	1 (4)
Membership of religious community, n (%)						
Yes	56 (54)	13 (59)	10 (43)	12 (48)	5 (63)	16 (62)
Christianity	47 (45)	10 (45)	10 (43)	9 (36)	5 (63)	13 (50)
Islam	1 (1)	0 (0)	0 (0)	0 (0)	0 (0)	1 (4)
Not specified	8 (8)	3 (14)	0 (0)	3 (12)	0 (0)	2 (8)
No	43 (41)	7 (32)	12 (52)	13 (52)	3 (38)	8 (31)
Not specified	5 (5)	2 (9)	1 (4)	0 (0)	0 (0)	2 (8)

a Multiple entries were possible for marital status and professional situation.

b During the data collection period (August 2022 to April 2023), the average conversion rate was approximately 1 EUR = US $1.04.

### Data Collection

Based on an extensive literature review, a semistructured interview guide [40] (Multimedia Appendix 1) was designed by identifying the pressing issues regarding AI-CS in health (Figure 1, step 3). The key issues of control [41], consent [42,43], and human-machine interaction [44,45] were incorporated into the interview guide, resulting in 5 different sections (foundation, human-machine interaction, application example, control-related aspects, and consent-related aspects). The interview guide was discussed with other researchers and pilot tested, resulting in the addition of 2 practical examples of the use of AI in health (stress monitoring and palliative care). Due to the abundance of collected data, this paper mainly focuses on the sections relating to the foundation, human-machine interaction, and application examples.

Two questionnaires were used as additional material: the Hospital Anxiety and Depression Scale (HADS) questionnaire [46] was assessed to rule out the possibility that attitudes are influenced by anxiety or depression and a sociodemographic questionnaire (Multimedia Appendix 2) to avoid sampling bias.

The procedure was as follows: the day before the interviews, participants received written information about the study by mail and completed the 2 questionnaires (using Unipark software [Tivian XI GmbH]) [47]. Before the interviews started, the interviewers (EMH female, HB female, and DS male) explained the setting of the study and asked for the participants’ informed consent for the audio recording. Participants could choose to be interviewed on-site, remotely, or by telephone to ensure the representation of different age groups and levels of technical knowledge. All interviews were conducted between August 2022 and April 2023 and were pseudonymized and transcribed [48].

### Data Analysis

The interviews were analyzed using qualitative content analysis [35] in their original language (German or English). The techniques of content-related and scaling structuring were applied using ATLAS.ti software (ATLAS.ti Scientific Software Development GmbH) [49]. We combined deductive category assignment to refer to the current state of research with inductive category formation to consider the insights highlighted by the participants (Figure 1, step 5). We assigned three deductive categories: (1) attitudes, including the subcategories feelings (codes: positive, negative, and mixed feelings), thoughts (codes: opportunities, challenges, and values), and behavioral aspects (codes: experiences, implications, and preferred actions); (2) different measurement technologies*,* in order to be able to compare the above-mentioned forms of contactless sensor technologies with other contactless measurement technologies such as magnetic resonance imaging and X-ray and contact measuring devices such as wearable sensors; and (3) AI in order to gage the participants’ understanding to differentiate attitudes toward contactless sensor technologies and data analysis processes.

**Figure 2.**
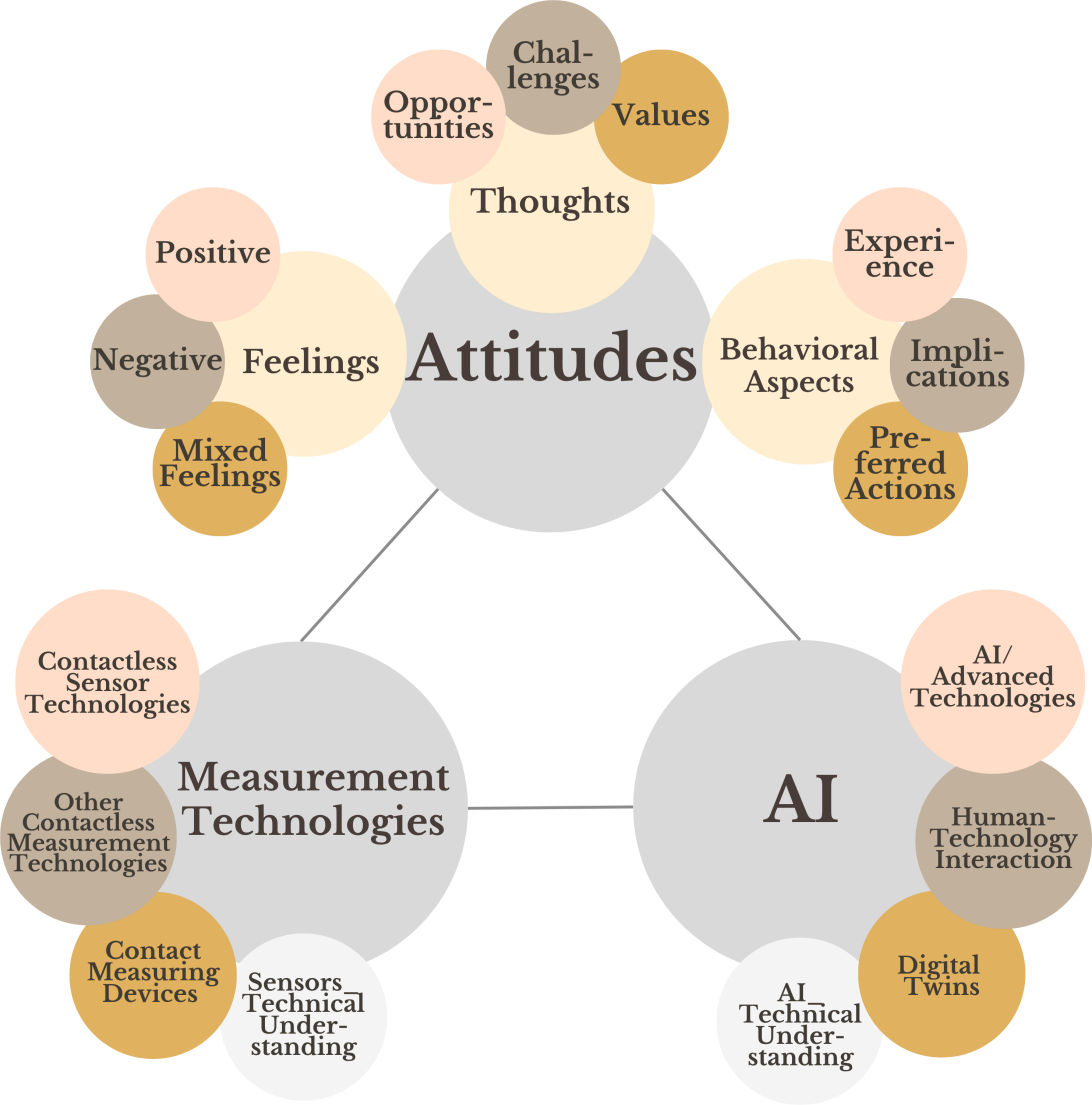
Code scheme. AI: artificial intelligence.

Based on 10 interviews (2 from each stakeholder group), the initial coding scheme was extended by the interdisciplinary, gender-mixed coding team (EMH [female and theology], LG [male and theology and physics], and CS [female and philosophy]) through the inductive formation of subcodes (Figure 1, step 6) within the deductively derived codes of the categories of feelings, thoughts, and behavioral aspects. The resulting coding scheme (Figure 2) was discussed with the wider research team (including experts from psychology, medicine, and computer science and reflecting researchers’ positionality) and refined in a peer debriefing. To identify possible discrepancies, the coding team carried out a parallel coding of 10 interviews, resulting in 95% coding consistency. All 104 interviews were then coded by 2 coders each, and discrepancies were discussed in biweekly meetings.

To report the findings, the first author (EMH) carried out a forward translation [50] of the German quotations, which were double-checked by a second researcher (AM) with a teaching qualification in English. In the results, quotations are cited by indicating the initial letter of the stakeholder group (P for patients, H for HCPs, R for researchers, PS for political stakeholders, and G for general public) and a number (the first number indicates an interview number out of 104 interviews, and the second number represents the code number). All results are visualized in an online tool called SENSAI [51], using the Graph View plug-in of the Obsidian software (Dynalist Inc) [52], which enables readers to access the relevant quotes themselves.

## Results

### Participants’ Characteristics

A total of 104 participants, aged 20 to 87 years, were included in our interview study, 55% (57/104) of whom were female. Table 1 depicts the characteristics and sociodemographic status of all participants. The participants were asked to assign themselves to a group: 22 identified themselves as patients, 23 as HCPs (ie, working as physicians, medical assistants, care specialists, or nurses), 25 as researchers (eg, in computer science and engineering, psychology, radiology, or law science), 8 as political stakeholders, and 26 as members of the general public. A total of 8 participants showed a critical value on the HADS. As these individuals were evenly distributed across the 4 stakeholder groups, no clear correlation can be drawn.

Interviews were conducted in German (n=98) or in English (n=6) on-site at the university building (n=11), remotely (n=89), and by telephone (n=4) and lasted between 23 and 58 minutes.

#### Feelings

**Figure 3.**
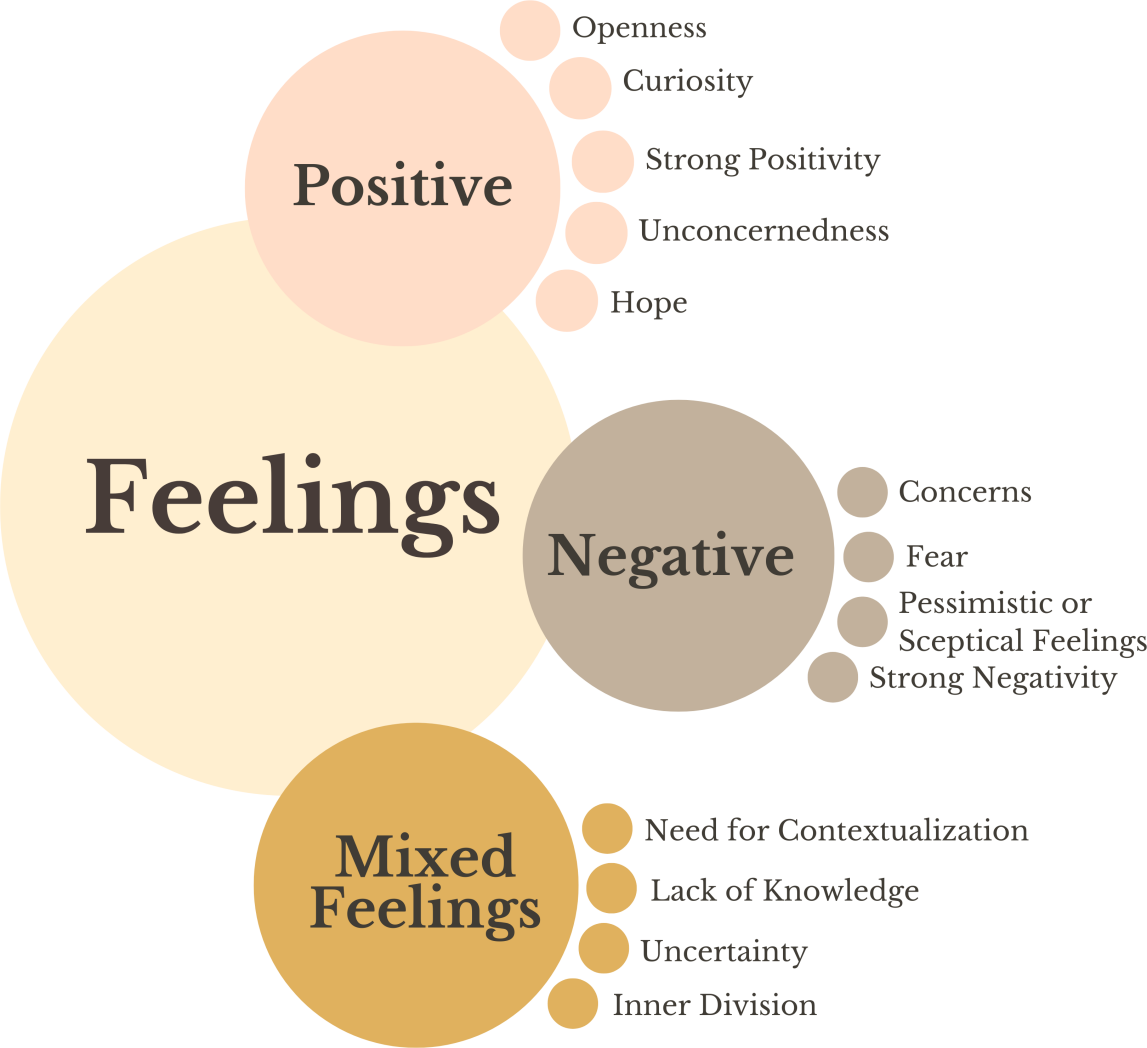
Overview of feelings identified by different stakeholder groups.

Feelings were first coded deductively into positive, negative, and mixed feelings*.* Second, inductive subcodes were added within each of these codes (Figure 3).

#### Positive

Participants expressed a great openness toward AI-CS (eg, PS97:59) (; Multimedia Appendix 3). In addition to the willingness to engage with the application, different forms of curiosity were expressed: the overall interest in sensors and their technical possibilities (P91:76, H83:26, R31:48, PS97:73, and G48:106), the specific expectation of advantages with personal or general benefits (P96:30, H74:54, R46:165, PS100:48, and G64:211) or hopes for the improvement of personal or general circumstances in the clinical context and health care (P58:31, P70:190, H74:54, PS102:153, and G7:260). Strong positivity was expressed in all stakeholder groups (eg, G4:72) and linked to individual experiences, “I have never had any negative experiences […]” (G37:96). Some participants mentioned their unconcernedness about using sensors in health (PS100:83), “[…] I don’t see any problem with that at all” (P79:45).

Notions of hope were expressed as positive feelings, with particular findings in the different groups. Patients hoped that the use of AI-CS could have an immediate (P45:87), but also long-term benefit (P9:130). With regard to treatment, patients saw no drawbacks of contactless over other sensory systems as long as it is conducive to successful treatment (P22:107). HCPs also expressed their hope (H83:161), but with a strong focus on the application in treatment and care (H39:150). Researchers rarely expressed their hope (R25:13), as they did not perceive any substantial difference between contactless and contact sensors, “this is simply […] a different sensor […]” (R16:19). Political stakeholders associated clear hopes with AI-CS in regard to their use in health in general (PS102:153) and in specific areas (eg, palliative care PS100:14). The general public rarely expressed specific hopes (G47:25); rather, curiosity about the sensor technology prevailed (G67:139).

#### Negative

In all stakeholder groups, concerns were particularly emphasized in relation to AI-CS (“problematic” P9:237; “difficult” R25:9, H85:11, G48:16; “would not feel comfortable” R38:32) (; Multimedia Appendix 4). Where these concerns were assumed as a possible outcome, rejection occurred (“don’t need it” P35:115, “don’t want that” H44:22, “would reject it” R46:32, and “would fight against it” G3:123).

Aspects of fear (“discomfort” R38:32, “worrying” PS101:28, and “not daring” G37:28) were mentioned less frequently, and either regarding specific developments (“I’m afraid this will eventually replace me […].” H74:155), partial aspects (“Because I’m […] a bit afraid of this electrosmog.” P94:7) or the technology as a whole (“Sometimes I find that frightening.” H66:110).

Pessimistic or skeptical feelings were expressed regarding the potential future effects (H28:12, G20:65, and PS101:138) or the technical feasibility, “[b]ut have we already reached the point where today’s data can be used to predict conditions in a few years’ time?” (P22:93). HCPs, in particular, raised fears that related to, among other things, the possible replacement of their own workforce (H74:155) or to the possibility of unconscious misuse (H66:110 and PS101:28) in sensitive environments (palliative care H75:41 and nonmedical H77:90).

Strong negativity occurred in the form of intense rejection, concern, or fear (“quite terrible” G23:142, “highly critical” H78:108, and “great danger” G24:71), relating to specific effects (H78:108 and R13:168) and areas: “[…] for something as vague as stress and depression, I find myself kind of flinching and thinking, oh, this is dangerous” (P95:23). The strong polarity within the stakeholder groups is particularly striking: the most frequent negative feeling expressed among patients was an associated potential for misuse (P73:32 and P91:64) as well as non–health-related use by the general public (economic G48:112, public G37:138, and work-related G23:20). The other stakeholder groups rarely came forward with strong negative opinions (H78:108, R46:32, and PS101:116), presumably due to a stronger focus on the application. It is striking that researchers expressed very few negative feelings.

#### Mixed Feelings

A strong need for contextualization came up in all stakeholder groups, referring to the type of sensor technologies (R15:138), the area of application (H83:31, PS100:28, and G18:15), and other effects (P96:31) (; Multimedia Appendix 5). Patients emphasized the need for contextualization (P56:24) also by noting that the attitude is strongly situational: “I don’t think anyone can say, oh, I’ll do it no matter what. It’s always a matter of the moment when the question is asked […] am I sure I’m comfortable with it or not?” (P70:107).

Related to that, there was a frequent emphasis on the lack of knowledge (“I can’t really imagine what they might be doing, because I can’t think of anything else to use them for, other than the practical examples from before.” P73:116) or experience (“And personally, I find it difficult to put myself in that position.” R46:152). Often, this was combined with a great level of uncertainty: “I haven’t made up my mind […] on that for now” (G59:58). Uncertainty was expressed, especially regarding the technical feasibility and the possible effects: “Uh, in stress research, it’s not really clear to me what’s going to come from […] knowing […] whether a person is stressed or not. […] I don’t have any idea yet how […] it will help us with diagnostics […]” (H88:55).

The aforementioned feelings resulted in an expression of inner division (“divided” G67:127, “ambivalent” R54:145, and “torn” H89:82) in varying degrees. HCPs put a strong emphasis on feelings of inner division between potential benefits (H75:3) and misuse (H66:193 and H72:34). Political stakeholders perceived the advantages of AI-CS, but contrasted them with possible challenges for the individual or society, “[s]o there are two hearts beating in my chest, you know? […] If you realize later that you could also do this and that cool thing with the data collected […] you really have to think very carefully about whether that really serves its purpose*”* (PS101:91). Members of the general public perceived a trade-off between collective medical progress and personal data protection (G18:15), also resulting in inner division (G67:127). Remarkably, patients expressed no inner division.

### Thoughts

Participants identified various opportunities, challenges, and values for AI-CS, often weighing different opportunities and challenges against each other. The range and breadth of opportunities and challenges identified by stakeholders were inductively categorized into medical, technical, economic, sociopolitical, and regulatory domains.

#### Medical Opportunities and Challenges

Medical opportunities and challenges were identified by all stakeholder groups in relation to different medical functions. Concrete medical challenges and opportunities according to the different medical specialties can be found in Multimedia Appendix 6.

All stakeholder groups highlighted the opportunities in terms of monitoring, diagnostics, treatment, prevention, and research as very important (Multimedia Appendix 7). First, respondents saw medical opportunities in improving (home) monitoring, particularly through continuous and long-term monitoring (R25:16). All stakeholder groups pointed out the benefits of improved patient self-monitoring in order to be better informed about their current situation and to develop a better sense of their own body (R50:19). Second, for diagnostics, this means that diseases and changes in the body can be detected at an early stage (R15:117). Third, by providing a better overview of the course of treatment*,* a more targeted treatment and better preparation for focus on patient consultations are enabled (H71:22). Fourth, participants mentioned that prevention could potentially play a greater role in health systems due to a changed understanding of health and disease (H74:141). Fifth, medical progress and research could be advanced, especially through individualization and the linking of contexts.

The aspect of potential harm to patients—through missed alarms despite medical urgency (PS103:72) in monitoring, through incorrect diagnosis or treatment—cuts across all challenges (Multimedia Appendix 8). With regard to monitoring, a researcher described their uncertainty about medical harm caused by data transmission (R27:11). In the field of diagnostics, “data that would be unproblematic [could] become a problem just by being collected” (H65:127). One researcher asked,

What is the normal range? What is outside? And there I think it is a bit critical to say or to define what is normal and what is abnormal. And maybe to reflect that back to people, that they are also in the abnormal range, and what does that do to people psychologically?

(R54:27)

### Technical Opportunities and Challenges

In terms of implementation, use, data, and advances in medical technology, various technical opportunities were identified (Multimedia Appendix 9). Implementation is facilitated by the easy and time-saving installation and the small size of the device (P45:10). The avoidance of cabling in general was highlighted, particularly by HCPs, as a great opportunity for intensive care, palliative care, patient transport, pediatrics, and dealing with older adults. The portable use of sensors enables automatic and continuous long-term measurement and the collection of comparative data outside of clinical hours: “[Y]ou know somehow, at the moment of the examination it suddenly doesn’t hurt anymore or it’s somehow a special situation” (PS99:12). Patient passivity eliminates missed measurements and typing errors (which can occur when information is entered manually): It’s “[…] just plug and play. It’s the best you can get. […] someone does the monitoring and cares for you in the background […]” (PS102:86). Other possible measurement distortions such as the white coat syndrome, unfamiliar measurement environments, touching the patient or study participant, and others are reduced or even avoided by the unconscious nature of the measurement (H65:5 and G4:106). In terms of the data generated, contactless measurement is perceived to contribute to more accurate, reliable, and objective data (R63:168). Four out of 5 stakeholder groups highlighted the potential to measure what cannot yet be measured, such as micromovements in joints, as a medical-technical progress. Patients, HCPs, and researchers described the possibility of measuring and detecting diseases based on large movements and movement patterns, but also small ones, such as tiny muscle movements invisible to the naked eye. The ability to gather information about conditions that previously could only be asked about verbally, such as anxiety, restlessness, and pain, is considered great potential (R34:44).

Across all challenges, all stakeholder groups placed particular emphasis on the technical challenges (Multimedia Appendix 10). Concerning the development of AI-CS, in particular, the set-up and the lack of technical expertise in using the sensors (eg, lack of digital literacy among older patients, H89:77) were considered to potentially pose problems for the implementation in health (H39:23). In terms of use, an important consideration was how to ensure that the device worked smoothly, without bycatch or interference (R38:20). At the same time, contactless sensors were described as having limited alignment capability (R32:107), and their accuracy and reliability were questioned several times (R43:1). With regard to the collected data, the significance of the data were described as limited due to the individuality of the parameters, which makes comparisons difficult (P73:172).

### Economic Opportunities and Challenges

It is striking that a common motif across all economic opportunities is savings of time, money, and resources (Multimedia Appendix 11). In terms of health systems, time savings through self-measurements at home, more efficient doctor visits, and cost savings through the early detection and reduction of treatment costs were mentioned. These opportunities are expected to relieve HCPs by making their work more efficient and easier, as there is no need for extensive training to operate the equipment (R38:37). In terms of product-specific economic opportunities, participants thought that contactless sensors could be produced and achieve widespread coverage more quickly due to the low cost of the devices (R15:43 and G53:93).

With regard to HCPs and patients, fears of overburdening, additional pressure, and extra work were expressed as challenges (Multimedia Appendix 12). HCPs would have to learn to use the systems intensively; system errors and incompatibility with other systems would mean extra work and tie up resources (G24:14), leading to a rubber band effect for HCPs (G61:110). Pressure on individual patients could increase, similar to the pressure on hospitals and health systems to reduce costs through behavioral change (G57:22). To cope with this pressure, hospitals could set up “sensor farms” (R46:25) to cut costs and use sensors without medical indication (P73:93). In particular, a possible increase in patient transparency vis-à-vis health insurance companies was seen critically (H41:21). Great fears were also expressed regarding the solidarity principle of public health insurance systems such as the German one, “whether [...] health services could not also be linked to certain lifestyles, [...] and that is actually an imperative that is categorically ruled out in Germany” (G57:54).

### Sociopolitical Opportunities and Challenges

Social opportunities (Multimedia Appendix 13) were only touched upon, mainly by researchers: health in general is increasingly taking center stage, and the societal benefits outweigh this. In addition to being accessible to the masses (R27:23), the use of sensors could contribute to “[…] be more mindful, especially if you [...] do different training sessions or exercises or just know [...] what your body needs [...] and maybe the togetherness is more balanced because you are in control of yourself” (G59:76). It can also reduce the burden on relatives, both by providing a useful measurement tool and by relieving them of the need to be present during disease-altering events (R13:143).

Particular political opportunities (Multimedia Appendix 13) are to overcome barriers (such as language, or to sustain accessibility in the patient’s room; P80:67) and to reach people who avoid hospitals because of fear (H83:61). One political stakeholder described the use of sensors for immobile patients as an opportunity to address demographic change despite the shortage of physicians (PS104:80).

Identified social challenges (Multimedia Appendix 14) include various types of fears (of surveillance, loneliness, staff cuts, and others) and risks to individuals. More direct access to one’s own data could make patients feel anxious and discouraged and quickly lead to overfocus: “[P]hysical health even more steps out of the private sphere [...] and becomes an object [...] of public display. [...] [T]his display of inner strength, of health and vitality, also creates pressure [...] on people who are unable to do so because they may simply be ill” (G57:2). In terms of interaction, a possible influence on social development (R46:179), on social participation (R76:22), and an increasing lack of physical contact were mentioned. In relation to human-human interaction (H74:44), a patient described the importance of human contact from their own experience: “I managed to get back on track to a certain extent. But only with the human element. With technology alone [...] I would [...] no longer see any sense in life” (P69:85). ‬‬‬‬Several participants highlighted the role and importance of touch in the healing process: “[Y]ou can also simply reassure people, establish contact with people […] by simply touching them” (H75:104). Contactless data collection often leaves a feeling of unreliability or a feeling of not having been properly examined (R15:139 and R43:24). Some participants drew parallels with the contactless situation during the SARS-CoV-2 pandemic (H74:71 and H86:103).

Low levels or lack of acceptance were identified as a political challenge (Multimedia Appendix 15), which may be due to the human impulse to initially reject the unknown (PS97:18), the presumed slow build-up of acceptance among patients and HCPs (PS102:43), and societal reservations about radiation (R8:127 and H92:47). One patient suggested that “[...] a discussion should only take place on a scientific level, [...] if the general [...] population takes part in a discussion, um, the topic is already lost anyway*”* (P68:40). In terms of preimplementation issues, the benefits of contactless measurement were questioned in general, not just for patients who were already wired (R15:46 and PS102:55). The judicious use of AI-CS was described as a challenging balancing process. The resources needed to operate the technology should not be wasted (G52:99). Political stakeholders in particular saw implementation challenges, for example, due to the slowness of change in the medical field (R30:118). Another challenge is also a not yet realized “[…] paradigm shift, which then favors a preventive […] health protection, health care” (PS102:55). Further ambiguities exist in the exact procedure (PS101:78).

### Regulatory Opportunities and Challenges

Only regulatory challenges (Multimedia Appendix 16) were identified by the participants. Several challenges related to consent—mainly mentioned by HCPs, researchers, and the general public—focused on the unobtrusiveness of AI-CS (R12:3). Consent was emphasized as all the more important, especially for radar-based technologies (G64:36 and R31:13), because of the greater safety risk of measuring people without their awareness (compared with contact measurement technologies G61:112). All stakeholder groups identified a high risk of abuse of the sensors (eg, stalking or surveillance in public spaces or by employers [PS101:89 and R31:120], or for use in a state sanction mechanism [R31:98]), even leading to dystopian fantasies of total surveillance (G47:5). Challenges were also described for the management of the collected data, that is, how individuals could retain control over and manage their data (H92:62), while at the same time ensuring data security (R32:96), privacy (R36:132), and transparency about their own data (R63:97 and G64:162). Regarding regulation, a “regulatory hole” (PS40:93) was addressed. Medical device approval was described as challenging (PS40:124 and PS102:124). Another problem identified was the reactive nature of the law, which does not provide a framework for future settings (PS102:60). Contrary to the political stakeholders, an HCP mentioned that they believe that legal and ethical rules would not prevent misuse (H66:64 and H85:25).‬‬

In summary, the analysis revealed a wide range of perceived opportunities in medical, technical, and economic terms. Opportunities, such as the impact on society, were mainly focused on by researchers and political stakeholders. Naturally, HCPs and patients focused strongly on the application and effects of AI-CS in health. The broad range of challenges mentioned across all stakeholder groups showed the perceived ambivalence of AI-CS (eg, in the technical implementation and data generation), leading to the need for good communication, preventive governance, and the overall importance of human interaction in the health system.

### Values

Trust, legal certainty, sociality, autonomy, individuality, and usefulness could be identified as core values of the different stakeholder groups towards AI-CS in health.

All stakeholder groups emphasized the need to build trust in the use of AI-CS in health, particularly with regard to the need for transparency, reliability, accuracy, and proven usability: “[…] if […] it is transparent beforehand what will be done with it, of course I would be in favor of that” (R30:26). Patients referred to the trust regarding the implementation of contactless sensors and AI, which is seen as “[...] a result of the relationship of trust that has developed between the patient and the doctor” (P22:96). Trust in these technologies is not yet there due to their recency, so it needs to be built: “There is still no basis of trust because you don’t know it yet [...]” (P73:119). Some patients also expressed a leap of faith in new technologies, particularly relating to their expectations of better health care or due to a perceived objectivity of the technologies, that is, seeing AI as a form of “objective value” (P9:261). On the other hand, HCPs took a comparatively matter-of-fact approach for formulating the conditions for trust, demanding scientific evidence, explainability, and validated usability of new technologies. HCPs also addressed the pitfalls and consequences of changing trust attributions and perceptions of AI for the individual doctor: “[D]o I trust the person who’s been doing this somehow for years, or do I trust the AI that has all my data?” (H78:57)

Second, the need for legal certainty for the acceptance of new technologies was identified across all stakeholder groups—in particular for the authorization of new technologies, but also for the secure storage of data that are not accessible to third parties (“neighbors” G60:59).

Third, different stakeholder groups emphasized the importance of human interaction, humanity, and human connection, as well as care in health, summarized as sociality. Patients feared that the central relational aspect of being human would be lost using contactless sensors (H83:40). Contactless sensors would lack empathy—that’s why some participants would prefer to have a human in the loop who “[...] is somehow able to absorb even more than a sensor” (P95:44).

Fourth, autonomy was identified as a crucial value, with stakeholders underlining the importance of self-control and decision-making in the context of health (G64:40). This value was further embraced by the notion of freedom to make an informed decision, including the choice not to know certain pieces of health-related information. The unpredictability of the future, together with an openness to various outcomes and the fragility of life, are some of the values that were articulated across all stakeholder groups (eg, G57:44).

Fifth, the recognition of individuality came up as a topic of conversation, particularly in interviews with patients, HCPs, and the general public. Concerns were raised about the potential for contactless sensors to overlook individual differences: “Oops, I’m no longer an individual here, I’m […] this biological entity, a human being. And one is like the other. And I don’t like that” (P22:63).

The sixth value mentioned across all stakeholder groups was usefulness, particularly in terms of medical usefulness for a patient’s individual health or for the medical-technical progress in general (H92:102). One researcher mentioned:

[…] I would certainly also benefit from the research results […] through other people. In the same way, I would participate in order to advance research. To steer the progress of knowledge.

(R38:29)

### Behavioral Aspects

#### Experience

Specific experiences with contactless measurement technologies in a medical context were barely described across all groups (eg, P68:27). Detailed reports of experiences with AI-CS came only from researchers (R32:138 and R38:246). At the same time, however, there were numerous analogies to the operation of the technology, which can be divided into 2 main observations: first, a link was made between the way sensors work and human capabilities (eg, P73:15). The human capacity to evaluate internal states without physical touch is thought to be reinforced and/or substantiated by AI-CS. Secondly, analogies were drawn to existing analog technologies that measure from the outside without contact, for example, X-rays (P73:122).

Another important role played by the question of whether the use of AI-CS leads to more (perceived) supervision, which, however, was contested by the experience of HCPs (H92:64). In addition, some researchers pointed out that the patient’s previous experiences were significant:

If someone has ever been in intensive care [...] and was completely wired up for a fortnight and had to press the bell every time, […] they will immediately say, [...] I want to be monitored [...] contactlessly [...].”

(R42:63f)

#### Implications

Three implications were raised. The first one concerns changes in the doctor-patient relationship caused by new contactless measurement technologies. Some anticipate changes in the level of monitoring, which could lead to uncertainty and sometimes even anger (P87:88), while HCPs described the developments as a logical progression toward further personalization of medicine (H39:132). Researchers expressed similar sentiments: “Why should it suddenly be any different with contactless sensors if it happens in a medical setting […]” (R76:70)? Nevertheless, conditions for a successful introduction of the sensors, such as transparency and education (eg, PS103:31), individual medical background and attitude (eg, PS104:30), as well as serial application (PS102:89) were emphasized particularly by political stakeholders. One participant of this group assumed that patients’ sense of surveillance will be much less in a medical context than when the sensors are used in private homes or for other purposes (PS100:22). This is consistent with the also mentioned assertion that the medical benefits are likely to outweigh any legal concerns (PS102:66).

The second implication relates to shifts in the values recorded. In particular, the question of possible changes in the conception of trust plays a central role here. Some respondents seemed to trust in the prevention of misuse and general regulation by legal measures (G37:90). This especially means that decision-making authority remains with the HCPs:

And if the data collection from the contactless sensors has shown that, then the doctor will say: ‘[…] now we’ll do it like this.’ Then I would have so much confidence in the doctor that they can interpret what the AI says […].

(G47:81)

A third implication concerns anthropological issues. In addition to the ascribed analogy between the functioning of the sensor and human abilities, the discussion also centered on how the use of contactless sensors is changing the understanding of what it means to be human. With the elimination of touch as a basic element of measurement, the loss of its significance as a deeply human need is presumed (eg, H86:103). AI-CS is also expected to have an impact on self-interpretation techniques and experiences of efficacy: “Yes, I’m quite convinced of that. In the same way that people have forgotten how to find their way around since there are navigation systems […] and so on” (P95:57).

#### Preferred Actions

The preferred action relates to 3 main areas: first, the impact of AI-CS on forms and ways of establishing practices of consent was discussed. Some participants seemed to be largely indifferent: “But if the doctor explains that we have now come so far as to be able to make the whole thing contactless from other, uh, indicators, uh, that can be obtained from other bodily functions, then I can say: Well, fine, please” (P22:163), while others were rather positive (P70:114). Although AI-CS may change the established routines of giving consent, the importance of this consent is strongly emphasized (H41:30 and R65:16). As all groups thought, it is important for patients to know when a measurement is being taken and when it is not.

A second area concerns the assessment of (institutional*)* responsibility. Here, the prevention of abusive data collection was a rather important issue. Political stakeholders focused strongly on responsibility and its precise organization (eg, PS40:153), distinguishing between different levels of moral and legal responsibility, affecting both manufacturers and HCPs (eg, PS97:89). One political stakeholder argued that responsibility lies with the manufacturer and the user (PS40:153), but that the explicit approval of the patient must be taken into account (PS40:24). It was considered crucial, especially by HCPs, that AI-CS be embedded and evaluated in institutional decision-making pathways (H44:36 and H75:6). The use of these sensor technologies should intend to support staff, not replace them (H28:22 and G67:95). In addition, further research and institutionalized monitoring should ensure the functionality and safety of the sensors (G67:131). Furthermore, within the area of responsibility, all groups saw education as an important institutional aspect (P35:17, H26:10, R19:47, PS102:40, and G5:31): There is a need for training in the skills required—not only to explain the new measurement techniques, but also to understand their implications and limitations.

A third area of preferred action concerns the question of whether new measurement technologies will lead to the medicalization of everyday life. In other words, health issues also play a role in areas where they are not (or should not be) the focus of attention. Very different assessments of such a possible extension emerged: “Yes, so perhaps not […] in working life, in the office” (P45:122). In contrast: “Well, I don’t know if I’m raving too far or thinking too high, but for me it would be ideal to have such a sensor in my pocket, day and night, really”, (P58:31).

## Discussion

### Brief Summary

The study addresses 2 key shortcomings: the lack of empirical research on attitudes toward AI-CS and the lack of a comprehensive stakeholder approach.

Regarding the first shortcoming, the results go beyond other studies by providing a comprehensive picture of attitudinal aspects toward AI-CS by following the analytical 3-component model. Overall, the results show a high level of openness to AI-CS in health across all stakeholder groups. Besides, 2 trends emerge from the results: first, greater experience and knowledge correlated with a reduced tendency to react emotionally. In particular, researchers were less likely to express strong emotions, both negative and positive, while HCPs showed little to no strong negativity. Both groups tended to take a sober view, with a focus on realistic expectations. In contrast, those with little or no experience or knowledge of the technology expressed stronger attitudes (showing both very enthusiastic, very uncertain, and/or negative feelings) toward certain aspects of the technologies.

Second, participants with good experiences with technologies were generally more open and positive toward contactless sensor technologies. This finding is, on the one hand, supported by previous research linking attitudes to prior experience [53]. On the other hand, it highlights the observation that attitudes are very likely to be based on the perceived social changes associated with a new technology, which is in line with similar empirical studies on people’s attitudes and values when interpreting emerging technologies [54-56]. This observation also backs the theoretical assessment that AI should not be viewed as an abstract technical variable, but rather in terms of sociotechnical systems [57-60]. In this sense, AI-CS does not represent a completely new case, but rather raises issues that are inherent to human-technology interactions in a similar way to other AI-driven technologies used in health, such as issues of trust [61].

Regarding the lack of a comprehensive stakeholder approach, the study offers an innovation pipeline approach and responds to the current debates on AI that call for the inclusion of diversity and multiplicity of stakeholders to avoid harm [62]. Furthermore, it explicitly “[…] account[s] for the potential limitations of one group speaking for another group, which may in fact disagree” [63]. Different particularities emerged between the groups: patients showed an altruistic moment of great willingness with regard to sharing data if they see a medical benefit, while political stakeholders put an emphasis on practical implementation and suggested concrete proposals by taking into account current regulatory developments. At a thematic level, all stakeholder groups identified technical opportunities, but patients, HCPs*,* and researchers provided more detailed insights, reflecting their direct involvement in the development, evaluation, and application of sensor technologies. The multistakeholder approach ensures that different perspectives are well-represented. The sample size of 104 participants allows commonalities and divergences across all stakeholder groups to be highlighted. Despite some overlap between groups (eg, between patients and the general public*,* or HCPs and researchers), self-classification facilitated nuanced insights from each stakeholder group.

### Implications for Governance and Policy

Two key lessons emerge from the findings: first, providing clear and targeted information and facilitating (nonmedical) hands-on experience with AI-CS can play a crucial role in mitigating rejection. Understanding of the term “contactless” emerged as a key factor in shaping attitudes, suggesting that targeted information could address misconceptions effectively. At the same time, the diversity of associations highlighted by all stakeholder groups points to the overarching importance of attitudes over detailed technical knowledge. While technical understanding may reduce initial uncertainties, acceptance of AI-CS is ultimately influenced by broader attitudes and the ideas that participants associate with them.

In our study, 3 tensions were shown to be important: first, the tension around contact (lessness) and the importance and ambivalence of touch; second, the tension between protection and surveillance in relation to the path- and context-dependency; and third, the tension between the benefits and challenges of unobtrusiveness, particularly in relation to control and governance implications.

Starting with the first tension, the concept of “contactless” revolves around the question of whether or not one is being touched. All stakeholder groups identified this question as crucial, showing an ambivalence about the importance of touch and the potential benefits of avoiding it: on the one hand, touch and especially being touched were described as a deeply human experience that was considered an integral part of medical consultations, especially by HCPs. Touch is considered essential in the relationship between people, but also in the relationship with oneself, one’s body, and its capabilities. A contactless sensor may not be able to capture all parts of the (bodily) self, but will be limited to quantitative parameters, which in turn will limit the understanding of a healthy and a sick body. This could eventually lead to a problematic image of the body as always potentially ill. It resonates with wider debates about the quantification and datafication of health, where complex, embodied experiences risk being reduced to measurable parameters [64]. Such reductionist tendencies are particularly relevant in sensory and embodied health care [65], where touch plays both diagnostic and relational roles.

On the other hand, the absence of touch was seen by all stakeholder groups as an important way of avoiding unwanted intimate physical contact, potentially removing one of the barriers to seeking medical advice. Beyond this practical dimension, participants agreed that the recording of symptoms and parameters is not mere measurement, but also involves an anthropological dimension. To be measured means to be under the gaze of an “other.” This interaction shapes the understanding of what it means to have an experience of one’s own bodily experience classified and interpreted from the perspective of another. The use of technologies such as contact or contactless systems introduces additional layers of complexity to this notion. For example, when a cable is used to measure parameters, a physical boundary is established between a person’s body and its environment. In contrast, contactless technologies challenge traditional notions of where these boundaries lie. This highlights the sociotechnical dimension of such innovations in that it is necessary to renegotiate where the boundaries of the human body are seen [66,67].

Second, all stakeholder groups pointed to a particular path- and context-dependency and its significance for attitudes by drawing a clear distinction between health data collection within a defined institutional context and outside of it, marking a strong tension between protection and surveillance. The use of AI-CS within institutional health contexts (such as hospitals or doctors’ surgeries) was identified as a helpful tool for protection by facilitating the need for constant monitoring. The ability to monitor without restricting movement was seen as one of the key benefits of contactless automated systems, particularly for people with dementia or in fields such as palliative care. Outside of health and institutional settings, all stakeholder groups raised concerns about illegitimate monitoring and surveillance, potentially leading to a loss of control or even knowledge of one being monitored. Concerns about a strong intrusion into privacy were repeatedly highlighted, as well as the potential for harmful effects on both the individual and society [68]. This tension between protection and surveillance is a major challenge for the use of AI-CS, as the boundaries between these contexts are becoming increasingly blurred by the ability and flexibility to use them in everyday life and in different locations due to the portability of the devices.

Related to this, a third tension emerged around the benefits and challenges of the unobtrusiveness of the measurement using AI-CS. While some researchers highlighted the unobtrusiveness of the data collection as a major advantage in minimizing measurement distortions (eg, movement changes due to measuring devices attached to the body), other researchers and HCPs expressed concern that the use of AI-CS could lead to a loss of visibility and thus an important basis for control [41]. These different perspectives highlight the dual nature of invisibility as both a technical strength and a potential source of institutional challenges. To mitigate the risks associated with unobtrusive measurement, it is essential to ensure the visibility of (interaction) interfaces. This aspect becomes even more critical when contactless sensor technology converges with automation through AI: automation can contribute to more efficient analyses of the collected data and can provide a more robust basis for decision-making in complex situations. However, the integration of AI-CS requires good interface design and careful consideration of innovation processes within institutional decision-making pathways in the clinic [69]. Moreover, defining benchmarks and criteria for defining the benefits and harms of technical systems requires particular scrutiny [70]. These criteria should be aligned with broader societal goals, aiming to provide a common good and contribute to the reduction of structural inequalities [71,72]. For example, tensions between individual benefits and benefits for the largest possible group need to be taken into account.

In this context, regulatory frameworks play a pivotal role in navigating the ethical complexities of AI-CS in health. Well-designed regulations are essential to balance the tensions between protection and surveillance and to address the challenges posed by unobtrusive measurement. Regulations such as the Medical Device Regulation [73] provide a basis for mitigating these tensions and preventing harm. The findings highlight the importance of proactive governance to ensure the ethically aligned implementation of AI-CS in health.

### Strengths and Limitations of the Study

This study provides comprehensive and nuanced insights into attitudes toward contactless measurement technologies in the health sector. By integrating perspectives from a broad range of stakeholder groups, it directly addresses the previously identified gap in research that often considers only a limited set of viewpoints. The use of both onsite and online interviews proved particularly valuable, as it enabled the inclusion of participants across a wide age spectrum and with varying levels of technological affinity, from highly tech-savvy individuals to those with limited experience with digital tools.

Recruitment efforts extended both within and beyond Germany. Although the majority of participants were based in Germany (n=97), a smaller share (n=7) came from other countries. While the study primarily focused on the German context, the findings nevertheless carry relevance for international settings and contribute to broader discussions surrounding the global development and acceptance of AI-based health technologies, provided potential cultural context biases are taken into consideration.

Despite repeated attempts to ensure balanced representation across all stakeholder groups, the study was unable to recruit an equal number of political stakeholders compared to other groups. This imbalance underscores an ongoing challenge in recruiting political stakeholders for qualitative research of this kind, which may be due to their smaller group size in general (compared to patients, for example) and their accessibility. Notwithstanding this challenge, it is important to acknowledge that the study included political stakeholders from both regional and national levels.

The participants’ level of education was higher than the average in Germany. This could be interpreted as either a selection bias or an increased opportunity or willingness of higher-educated persons to participate in studies.

Furthermore, a lack of clarity surrounding AI-CS must be mentioned. Participants frequently asked which applications could be considered contactless. They gave different interpretations of the term and pointed to a wide range of associations with the technology, based on their prior experiences (eg, prior illnesses and social events such as the SARS-CoV-2 pandemic), personal health and nonhealth experiences, knowledge, technical understanding, and imagination. Overall, the results show a lack of information and technical knowledge that led to uncertainty and discomfort for some participants. If such uncertainty is exacerbated, it could contribute to rejection, influenced by pseudoscientific reasoning.

### Conclusion

Our contribution to research on AI-CS is 3-fold: first, it provides the first comprehensive overview of attitudes toward AI-CS in health, addressing the moral values and sociotechnical practices. By analyzing the attitudes of 5 different stakeholder groups along feelings, thoughts, and behavioral aspects, the study yields nuanced insights that can be further explored in the SENSAI online tool [51]. Additionally, it presents a detailed examination of potential applications (eg, home monitoring for dementia) and potential users (from children to older adults; Multimedia Appendix 5).

Second, we have demonstrated the importance of exploring attitudes toward AI-CS, which have not yet been empirically investigated. The study provides a broad picture of different components of attitudes, as participants expressed enthusiasm, rejection, or indifference (feelings), acknowledged certain opportunities and challenges, and considered which values were touched upon (thoughts), based on their experiences, formulated implications, and preferred actions (behavioral aspects). The use of semistructured interviews allowed the participants to bring in their own experiences and draw parallels with other devices they are familiar with, as AI-CS are still relatively new technologies. Furthermore, by providing a clear, deductively derived concept of attitudes that paves the way for avoiding the fuzzy equation with the terms perspectives, values, views, and others, the study makes an important methodological contribution to AI ethics and social science research on AI-based technologies.

Third, the key tensions (regarding the role of touch and contactlessness in health, between protection and surveillance in relation to path- and context-dependency, and regarding benefits and challenges of unobtrusiveness, particularly in relation to control and governance implications) call for robust regulatory frameworks to prevent the misuse of AI-CS and promote acceptance, including targeted information and facilitation of hands-on experience.

Through these 3 contributions, the study aimed to address the 2 shortcomings of the lack of a multistakeholder approach and the lack of empirical analysis of a hitherto unexplored technology by applying the concept of attitudes and its analytical precision. The study provides important entry points into different areas of research and practice and acts as a springboard for further research on AI-based health technologies in general and AI-CS in particular, as it highlights crucial points regarding sensor development at different stages, data analysis, and application opportunities and challenges in different medical specialties and functions. The findings on acceptance and preferred actions will be particularly useful for political consideration and possible implementation processes that could advance the governance and regulation of sensor technologies. By implementing these findings, AI-CS could contribute to preventing patient harm and improving patient safety [74].

## Supplementary material

10.2196/75783 Multimedia Appendix 1 Interview guide.

10.2196/75783 Multimedia Appendix 2 Sociodemographic questionnaire.

10.2196/75783 Multimedia Appendix 3 Overview of positive feelings expressed by different stakeholder groups.

10.2196/75783 Multimedia Appendix 4 Overview of negative feelings expressed by different stakeholder groups.

10.2196/75783 Multimedia Appendix 5 Overview of mixed feelings expressed by different stakeholder groups.

10.2196/75783 Multimedia Appendix 6 Medical opportunities and challenges according to the different medical specialties.

10.2196/75783 Multimedia Appendix 7 Medical opportunities identified by different stakeholder groups.

10.2196/75783 Multimedia Appendix 8 Medical challenges identified by different stakeholder groups.

10.2196/75783 Multimedia Appendix 9 Technical opportunities identified by different stakeholder groups.

10.2196/75783 Multimedia Appendix 10 Technical challenges identified by different stakeholder groups.

10.2196/75783 Multimedia Appendix 11 Economic opportunities identified by different stakeholder groups.

10.2196/75783 Multimedia Appendix 12 Economic challenges identified by different stakeholder groups.

10.2196/75783 Multimedia Appendix 13 Sociopolitical opportunities identified by different stakeholder groups.

10.2196/75783 Multimedia Appendix 14 Social challenges identified by different stakeholder groups.

10.2196/75783 Multimedia Appendix 15 Political challenges identified by different stakeholder groups.

10.2196/75783 Multimedia Appendix 16 Regulatory challenges identified by different stakeholder groups.

10.2196/75783 Checklist 1 COREQ checklist.

## Abbreviations

AI: artificial intelligence
AI-CS: artificial intelligence–based contactless sensors
COREQ: Consolidated Criteria for Reporting Qualitative Research
HADS: Hospital Anxiety and Depression Scale
HCP: health care professional
