# Supplementary material for: Exploring Attitudes Toward AI-Based Contactless Sensors in Health Among Five Stakeholder Groups: Qualitative Study
Source: J Med Internet Res. 2026 Apr 24;28:e75783. doi: 10.2196/75783 (PMC13108836; doi:10.2196/75783)
Supplement: Multimedia Appendix 1 [file jmir-v28-e75783-s001.docx]

**Qualitativ-empirische Untersuchung in „EmpkinS“:**

**Einstellungen zu KI-basierter Sensorik im Gesundheitsbereich**

**Einleitung und Erklärung des Ablaufs**

- **Vorstellung des*r Interviewer*in**
- **Vorstellung des Forschungsprojekts**
- **Vorstellung des Ablaufs**

**Vorbereitungen für das Interview**

- Abfrage der ausgefüllten **Fragebögen** via Unipark
- Abfrage des **Pseudonyms**

**Einwilligung für Audioaufnahme**

**Aufnahme starten!**

**Interview**

**Darstellung von 2 Anwendungsbeispielen**

1. Palliativmedizin: Berührungslose Überwachung der Herz- und Atemfrequenz
2. Stressforschung: Berührungslose Analyse der Körperhaltung
3. **Grundlegendes**
4. Welche Erfahrungen haben Sie schon mit berührungslosen Messtechniken gemacht, die berührungslos Informationen über den inneren Gesundheitszustand sammeln?
5. Sehen Sie Chancen oder Risiken, wenn im Gesundheitsbereich berührungslose Messtechniken eingesetzt werden?
6. Wie ist ihre Einstellung dazu, berührungslose Messtechniken im Gesundheitsbereich anzuwenden?
7. Welche rechtlichen Fragstellungen sehen Sie davon berührt?
8. **KI / Mensch-Maschine-Interaktion**
9. Verändert sich Ihre Einstellung, wenn für die Auswertung der erhobenen Gesundheitsdaten und für das bessere Verständnis von Krankheiten Künstliche Intelligenz eingesetzt wird?
10. Welche Veränderungen erwarten Sie durch die Interaktion von medizinischem Personal und KI-basierten Systemen im Gesundheitsbereich?
11. Welche Auswirkungen sehen Sie dadurch für sich?
12. Gibt es Bereiche, in dem sie die Interaktion von Menschen und KI-basierten Systemen im Gesundheitsbereich besonders befürworten oder ablehnen würden?
13. Was wünschen Sie sich für diese Interaktion?
14. Welcher Einfluss ergibt sich daraus für das soziale Miteinander?
15. Verändert sich Ihr Verständnis von Gesundheit bzw. Krankheit durch den Einsatz von neuen Technologien im Gesundheitsbereich?
16. **Anwendungsbeispiel**
17. Wenden wir uns einem Praxisbeispiel zu: Stellen Sie sich vor, Sie werden bei einem Arztbesuch mithilfe von Sensoren vermessen, oder Sie nehmen zuhause selbst Ihr Smartphone zur Hand und messen verschiedene Daten von sich. In beiden Fällen werden Ihre Daten gesammelt und zu einem digitalen Abbild von Ihnen zusammengestellt (z.B. um Ihre Herzfrequenz zu beobachten). Ihr Körper wird also digital simuliert. Daraus können Aussagen zu Ihrem jetzigen Gesundheitszustand getroffen werden, und zu Ihrem Gesundheitszustand in z.B. 10 Jahren. Was halten Sie davon?

*Bild: Digital Twins als weiteres Anwendungsfeld*

1. Wie würden Sie persönlich mit diesen Vorhersagen umgehen?
2. Was ist für den Umgang mit diesen Vorhersagen im Allgemeinen wichtig?
3. **Kontrolle**
4. Was verstehen Sie allgemein unter Kontrolle?
5. Was sind Ihre Anforderungen an Kontrolle im Gesundheitsbereich?
6. Welche Bedingungen müssen gegeben sein, sodass Sie den Einsatz von KI-basierter Sensorik im Gesundheitsbereich als kontrollierbar empfinden?
7. Wie wichtig ist es für Sie, über Ihre medizinische Behandlung Kontrolle zu haben?
8. Wer darf Kontrolle über Ihre medizinische Behandlung haben?
9. **Einwilligung**
10. Unter welchen Bedingungen würden Sie als Patient*in Ihre Einwilligung zum Einsatz von KI-basierter, berührungsloser Sensorik in Ihrer Behandlung geben?
11. Verändert sich für Sie Ihre Entscheidung, wenn berührungslose anstelle von berührenden Messtechniken im Gesundheitsbereich eingesetzt werden?
12. Welche Optionen wünschen Sie sich für Ihre Zustimmung?
13. Hängen die Bereiche Kontrolle und Einwilligung für Sie beim Einsatz von KI-basierter Sensorik zusammen?

**Ende**

**Bedanken** für die Teilnahme.

**Qualitative-Empirical Study in ”EmpkinS“:**

**Attitudes towards AI-based Sensors in health**

**Introduction and Explanation of the Procedure**

- **Introduction** of the interviewer
- **Introduction of the research project**
- **Introduction of the procedure**

**In preparation for the interview**

- Requesting completed **questionnaires** via Unipark
- Asking for **pseudonym**

**Asking for consent for audio recordings**

**Start of Recording!**

**Interview**

**Presentation of 2 application examples**

1. Palliative care: Contactless monitoring of heart- and respiratory rates
2. Research regarding stress: Contactless monitoring of posture
3. **Foundation**
4. What experiences have you made with contactless measurement technologies that collect information of your inner health without contact?
5. Do you rather see opportunities or risks in using contactless measurement technologies in health?
6. What is your position on using contactless measurement technologies in health?
7. What legal issues do you think could be concerned?
8. **AI/Human-Machine Interaction**
9. Will it affect your position if Artificial Intelligence is used for the evaluation of the collected data as well as for the better understanding of health issues?
10. What changes do you expect from the interaction of medical staff and AI-based systems in health?
11. What effects do you expect for yourself?
12. Are there areas in health where you would especially approve of or disagree with the interaction of human and AI-based systems?
13. What are your wishes for those interactions?
14. How could these interactions influence social interaction?
15. Is your understanding towards health and illness affected by the use of new technologies in health?
16. **Application Example**
17. Let’s look at a practical example: Imagine when seeing a doctor, you are measured by sensors. Or, when you are at home, you can collect certain data of yourself with your smartphone. In both cases, your data is collected and a digital image of yourself is created (to monitor your heart rate, for example). Your body will also have a digital simulation. This simulation could be used to analyse your overall health and predict your health status in ten years. What do you think of this?

*Image: Digital Twins as another area of application*

1. How would you personally deal with these predictions?
2. What is important, in general, for dealing with these predictions?
3. **Control**
4. What do you understand by the term “control“ in general?
5. What are your expectations of control in health?
6. What conditions are needed for you to experience the use of AI-based sensors in the health sector as controllable?
7. How important is it for you to have control over your medical treatment?
8. Who is allowed to have control over your medical treatment?
9. **Consent**
10. What requirements have to be met for you as a patient to agree to the use of AI based, contactless sensors in your treatment?
11. Will it affect your decision if contactless measurement technologies are used in health instead of ones that use physical contact?
12. What options do you wish for to give your consent?
13. In your opinion, are the areas of control and consent connected for the use of AI-based sensors?

**End**

**Thank you for your participation!**
