# Supplementary material for: Exploring Attitudes Toward AI-Based Contactless Sensors in Health Among Five Stakeholder Groups: Qualitative Study
Source: J Med Internet Res. 2026 Apr 24;28:e75783. doi: 10.2196/75783 (PMC13108836; doi:10.2196/75783)
Supplement: Multimedia Appendix 2 [file jmir-v28-e75783-s002.docx]

**Soziodemographischer Fragebogen**

1. Wie alt sind Sie?

[Offenes Antwortfeld]

1. Welchem Geschlecht fühlen Sie sich zugehörig?
   - weiblich
   - männlich
   - divers
   - Keine Angabe

[Einfachauswahl]

1. Was ist Ihr höchster Bildungsabschluss?
   - Kein Abschluss
   - (Qualifizierter) Mittelschulabschluss
   - Mittlere Reife
   - Fachabitur (Fachgebunde bzw. Fachhochschulreife)
   - Abitur (Allgemeine Hochschulreife)
   - Abgeschlossene Ausbildung
   - Fachschule/Technikerschule/Handelsakademie
   - Meisterprüfung
   - Bachelor
   - Master (Diplom, Examen, o.ä.)
   - Promotion
   - Keine Angabe

[Einfachauswahl]

1. Wie ist Ihr Familienstand?
   - Alleinstehend
   - In Partnerschaft(en) lebend
   - Sorgeverantwortung für Minderjährige
   - Pflegeverantwortung für Angehörige
   - Keine Angabe

[Mehrfachauswahl möglich]

1. In welcher beruflichen Situation befinden Sie sich gerade?
   - arbeitend
   - Arbeitsbeginn (erste 2 Jahre seit Arbeitsstart)
   - arbeitslos
   - Ausbildung/Studium
   - Jobwechsel
   - krankgeschrieben
   - Rente
   - Sabbatical
   - Umschulung/Weiterbildung
   - Sonstige: ___________________
   - Keine Angabe

[Mehrfachauswahl möglich]

1. Wie sind Sie krankenversichert?
   - gesetzlich
   - privat oder mit Krankenhauszusatzversicherung
   - sonstige: ___________________
   - keine Angabe

[Einfachauswahl]

1. Wie viel Geld geben Sie im Monat durchschnittlich für technische Anwendungen (z.B. Apps, Streaming-Abos, etc.) und Geräte aus?
   - Kein Geld
   - 1-10€
   - 11-25€
   - 26-50€
   - 51-100€
   - 101-250€
   - Über 250€
   - Keine Angabe

[Einfachauswahl]

1. Sind Sie Mitglied einer Religionsgemeinschaft oder fühlen sich einer zugehörig?
   - Ja: [Offenes Antwortfeld]
   - Nein
   - Keine Angabe

[Einfachauswahl]

**Sociodemographic questionnaire**

1. How old are you?

[Open answer field]

1. Which gender do you associate with?
   - Female
   - Male
   - Diverse
   - Not specified

[single choice]

1. What is your highest level of education?
   - No degree
   - Qualified secondary school leaving certificate
   - Technical baccalaureate or entrance qualification for universities of applied sciences
   - A-Level-equivalent (general university entrance-qualification)
   - Completed Apprenticeship
   - Technical college/technical school/trade school
   - Master craftsman’s diploma
   - Bachelor’s Degree
   - Master’s Degree (Diploma, State Exam or similar)
   - Doctorate
   - Not specified

[Single Choice]

1. What is your marital status?
   - Single
   - Living in partnership(s)
   - Custodian of a minor
   - Caregiver for relatives
   - Not specified

[Multiple answers possible]

1. In which professional situation are you currently?
   - Working
   - Just started working (first two years since start of work)
   - unemployed
   - Apprenticeship/Studying
   - Job change
   - On sick leave
   - Pension
   - Sabbatical
   - Retraining
   - Other: [Open answer field]
   - Not specified

[Multiple answers possible]

1. How are you covered by health insurance?
   - Public health insurance
   - Private or with supplementary hospital insurance
   - Other: [Open answer field]
   - Not specified

[single choice]

1. How much money do you spend on average per month on technical applications (e.g., apps, streaming subscriptions, etc.) and devices?
   - No money
   - 1-10€
   - 11-25€
   - 26-50€
   - 51-100€
   - 101-250€
   - Over 250€
   - Not specified

[single choice]

1. Are you a member of a religious community or do you feel you belong to one?
   - Yes: [Open answer field]
   - No
   - Not specified

[single choice]
