# Supplementary figures and images for: Exploring Attitudes Toward AI-Based Contactless Sensors in Health Among Five Stakeholder Groups: Qualitative Study
Source: J Med Internet Res. 2026 Apr 24;28:e75783. doi: 10.2196/75783 (PMC13108836; doi:10.2196/75783)

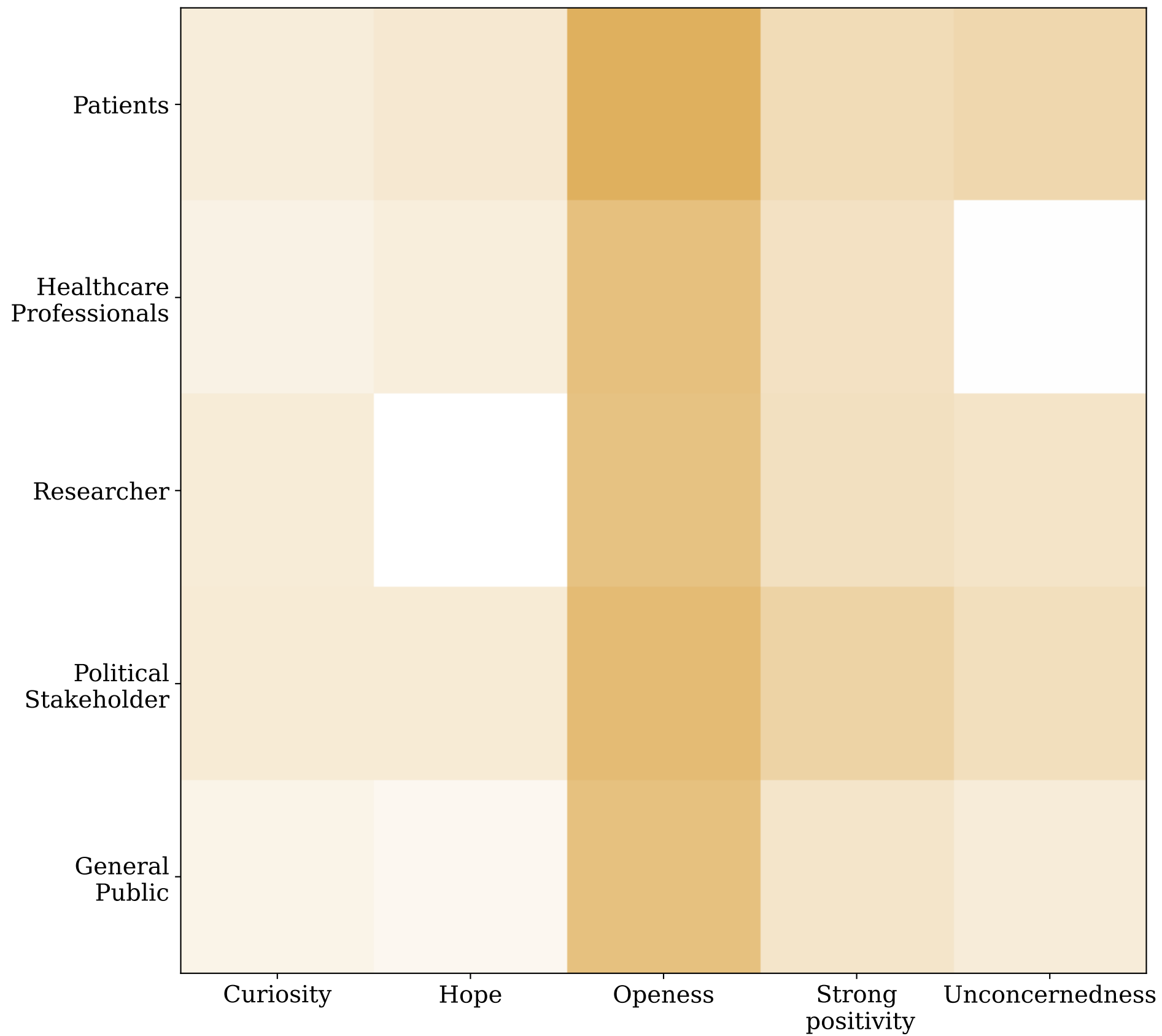

Supplement: Multimedia Appendix 3 [file jmir-v28-e75783-s003.pdf]

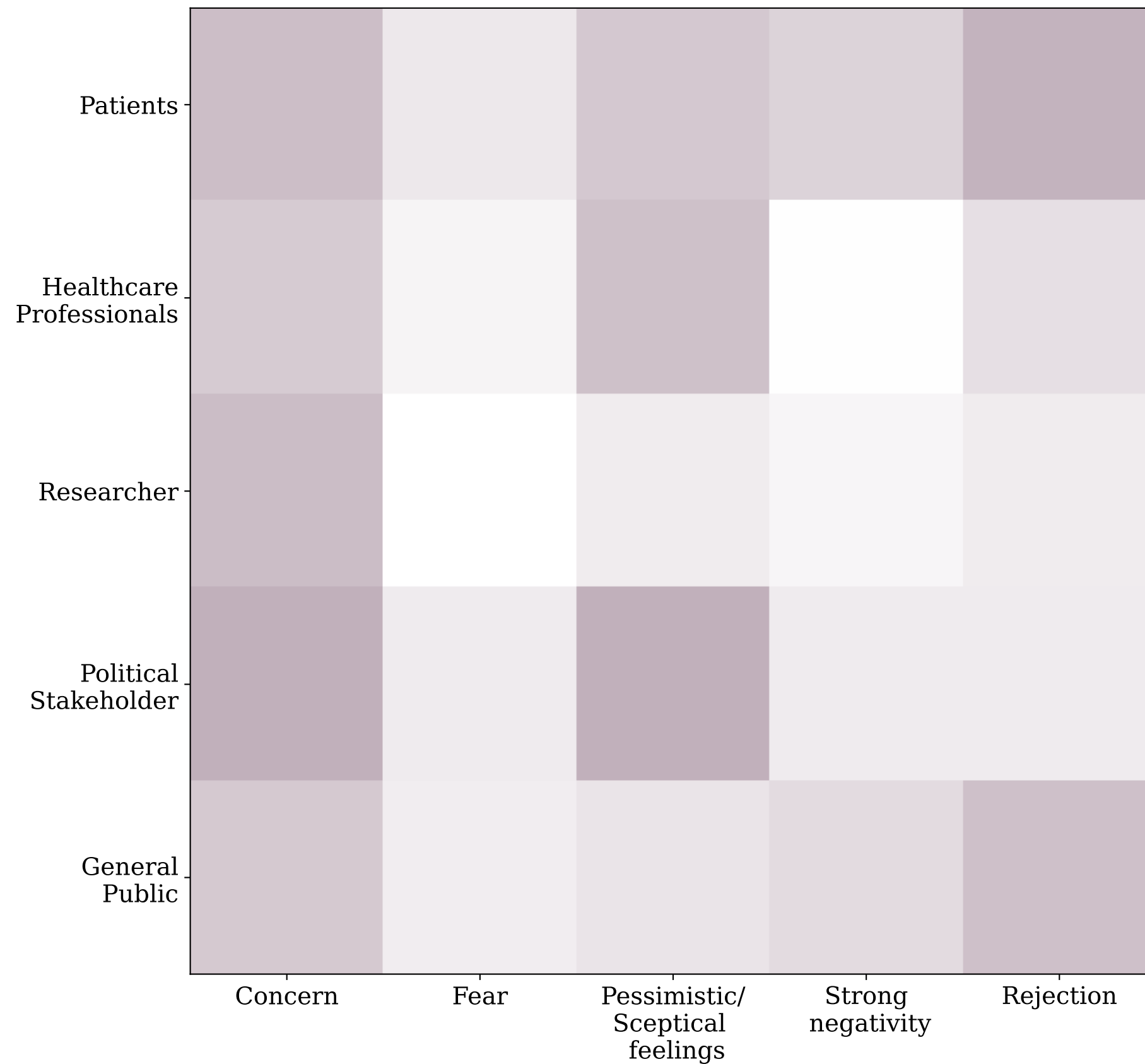

Supplement: Multimedia Appendix 4 [file jmir-v28-e75783-s004.pdf]

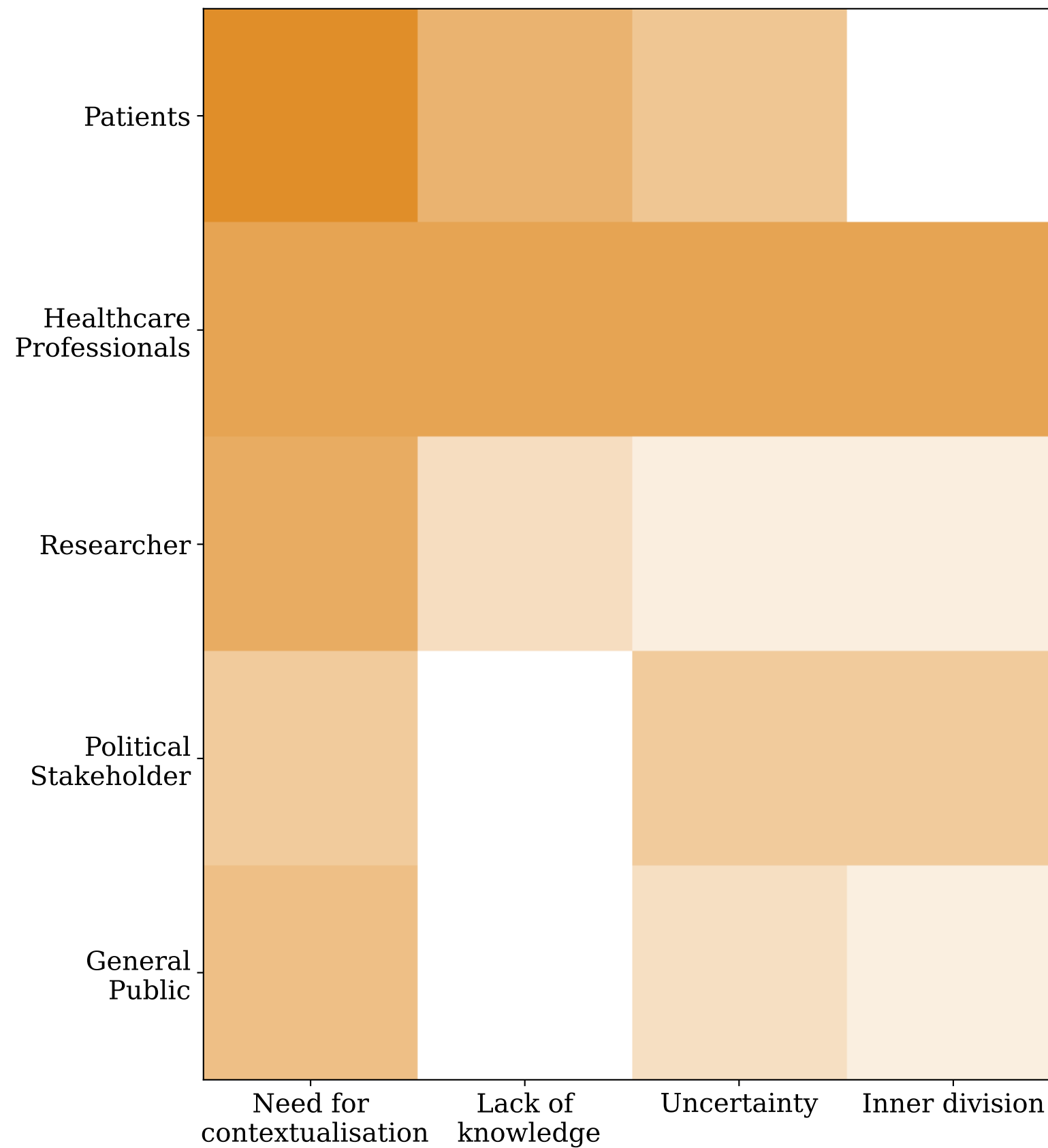

Supplement: Multimedia Appendix 5 [file jmir-v28-e75783-s005.pdf]
