# Supplementary material for: Exploring Attitudes Toward AI-Based Contactless Sensors in Health Among Five Stakeholder Groups: Qualitative Study
Source: J Med Internet Res. 2026 Apr 24;28:e75783. doi: 10.2196/75783 (PMC13108836; doi:10.2196/75783)
Supplement: Multimedia Appendix 6 [file jmir-v28-e75783-s006.docx]

| **MEDICAL SPECIALTIES: OPPORTUNITIES** | | Patients | Healthcare Professionals | Researcher | Political Stakeholder | General  Public |
| --- | --- | --- | --- | --- | --- | --- |
| **CARE** | | | | | | |
| Improving the care situation in general | | X |  |  | X |  |
| Facilitation in nursing homes and outpatient care | | X | X |  | X | X |
| Great potential for palliative care | | X | X | X | X | X |
| Increasing patients' safety | |  | X | X |  |  |
| Avoiding unwanted intimacy | |  | X |  | X |  |
| Possibility of accurate death prognosis | |  |  | X |  | X |
| **DERMATOLOGY AND VENEROLOGY** | | | | | | |
| Overall improvement of dermatologists' work | | X | X |  |  |  |
| **EXCEPTIONAL CIRCUMSTANCES (ICU/EMERGENCY MEDICINE)** | | | | | | |
| Great potential for use in emergency medicine | | X | X | X | X | X |
| Great potential for use in ICU | |  | X | X |  |  |
| Great potential for contactless pandemic monitoring | |  | X |  |  |  |
| **GYNAECOLOGY** | | | | | | |
| Whole-body health data collection | | X |  |  |  |  |
| Increased comfort for mother and unborn child | |  | X |  |  |  |
| **GENERAL PRACTICE** | | | | | | |
| Facilitating check ups | |  |  | X | X |  |
| Generation of information beyond the patient report | | X | X |  |  |  |
| Data generation in the waiting room | | X |  |  |  |  |
| Great potential for use in telemedicine | | X |  |  | X | X |
| **INTERNAL MEDICINE** | | | | | | |
| **Cardiology** | | | | | | |
|  | Long-term cardio data | X |  |  |  |  |
|  | More objective cardio data |  | X |  |  |  |
|  | Improve general cardiac data measurement (comfort, early diagnosis) | X | X | X | X | X |
|  | Heart attack alert | X |  | X | X |  |
|  | Informing the patient about unknown cardiac problems | X |  |  |  |  |
| **Endocrinology, diabetes, and metabolism** | | | | | | |
|  | Avoidance of skin irritation | X |  |  |  |  |
|  | Control diabetes patient based on constant data generated by sensor |  | X |  |  |  |
|  | Automatic blood glucose monitoring |  | X |  |  |  |
| **Nephrology** | | | | | | |
|  | Improved treatment (based on consistent, reliable data) |  | X |  |  |  |
| **Oncology** | | | | | | |
|  | Early diagnosis of cancer |  | X |  |  |  |
|  | More convenient check-up |  | X |  |  |  |
| **Rheumatology** | | | | | | |
|  | Control of patient behaviour | X |  |  |  |  |
|  | Alert function | X |  |  |  |  |
|  | More informed treatment |  | X |  |  |  |
|  | Early results in rheumatoid arthritis | X |  |  |  |  |
|  | Long-term rheumatology data |  | X |  |  |  |
|  | Diagnosis of chronic diseases | X | X |  | X |  |
|  | Pain reduction during measurement | X |  |  |  |  |
| **NEUROLOGY** | | | | | | |
| Great potential for neurology | | X | X | X | X | X |
| Early diagnosis of chronic diseases | |  |  | X |  |  |
| **OPHTHALMOLOGY** | | | | | | |
| Enabling objective and stable measurements | |  |  | X |  |  |
| **ORTHOPEDICS** | | | | | | |
| Motion capture | | X |  | X | X |  |
| **PAEDIATRICS** | | | | | | |
| Great potential for paediatrics | | X | X | X |  |  |
| Early diagnosis of chronic diseases | | X |  |  |  |  |
| **PHYSICAL MEDICINE AND REHABILITATION** | | | | | | |
| Monitoring of movements without the presence of professionals | |  |  | X |  |  |
| Objective measurement | |  | X |  |  |  |
| Enabling barrier-free accessibility | | X |  |  |  |  |
| **PSYCHIATRY** | | | | | | |
| Special application possibilites | |  | X |  |  |  |
| Availability of more objective measurement data | |  | X |  |  |  |
| Psychology | |  | X |  |  |  |
| Diagnostic support | |  | X | X |  |  |
| Relevance of sensory/body language measurement | | X | X | X | X |  |
| Early recognition of signs | | X |  |  |  |  |
| Individual stress reduction | |  |  | X |  | X |
| More meaningful data | |  |  | X |  |  |
| Awareness of own stress levels | |  |  |  |  | X |
| Reduction in medication use | |  |  |  |  | X |
| Less contact with doctors through technology | |  |  |  | X |  |
| **RADIOLOGY** | | | | | | |
| Special application possibilities | | X |  |  |  |  |
| Evaluating data for later decision support | |  | X |  |  |  |

| **MEDICAL SPECIALTIES: CHALLENGES** | | Patients | Healthcare Professionals | Researcher | Political Stakeholder | General  Public |
| --- | --- | --- | --- | --- | --- | --- |
| **CARE** | | | | | | |
| Challenging palliative care principles | | X | X | X |  |  |
| Limited/wrong prognosis of death (causing discomfort) | |  |  | X |  | X |
| **EXCEPTIONAL CIRCUMSTANCES (ICU/EMERGENCY MEDICINE)** | | | | | | |
| Unnecessary harmful resuscitation due to false alarms | |  |  |  | X |  |
| **GENERAL PRACTICE** | | | | | | |
| Negative impact on collected data due to patient stress | |  | X |  |  |  |
| **INTERNAL MEDICINE** | | | | | | |
| **Cardiology** | | | | | | |
|  | Missed medical events (e.g. heart attack) due to technical failure/unreliable data |  |  |  |  | X |
| **Endocrinology, diabetes, and metabolism** | | | | | | |
|  | False insuline medication |  | X |  |  |  |
| **Oncology** | | | | | | |
|  | Non-invasive measurement in oncology less reliable |  | X |  |  |  |
| **Rheumatology** | | | | | | |
|  | Inability to monitor muscles and joints | X |  |  |  |  |
| **MEDICAL GENETICS** | | | | | | |
| Difficulties in obtaining reliable data for predictions | |  | X |  |  |  |
| **NEUROLOGY** | | | | | | |
| Seizures (e.g. epileptic) not detected by sensors before they occur | | X |  |  |  |  |
| Risk of overdiagnosis | |  | X |  |  |  |
| **ORTHOPEDICS** | | | | | | |
| Parameters vary according to patient characteristics | |  |  |  | X |  |
| **PSYCHIATRY** | | | | | | |
| Patients' mistrust of technology in psychiatry | |  |  |  |  |  |
| Difficulties in measuring qualitative/subjective aspects of an illness (e.g. depression) | | X |  |  |  | X |
| Difficulties in distinguishing certain diagnoses by sensors (e.g. grief vs. depression) | | X |  |  |  |  |
| Risk of discovering diagnoses in people with anxiety disorders | |  |  |  |  | X |
| Parameters vary according to patient characteristics | | X |  |  | X |  |
| Risk of overdiagnosis/misdiagnosis | |  | X |  |  |  |
| Difficult to determine a "normal" value (without context) | | X |  | X |  |  |
| Risk of forgetting how to pay attention to the body's signals | | X |  |  |  |  |
| Increase patients' stress levels through the use of new technology | |  |  | X |  | X |
