# Supplementary material for: Exploring Attitudes Toward AI-Based Contactless Sensors in Health Among Five Stakeholder Groups: Qualitative Study
Source: J Med Internet Res. 2026 Apr 24;28:e75783. doi: 10.2196/75783 (PMC13108836; doi:10.2196/75783)
Supplement: Multimedia Appendix 7 [file jmir-v28-e75783-s007.docx]

| **MEDICAL OPPORTUNITIES** | Patients | Healthcare Professionals | Researcher | Political Stakeholder | General  Public |
| --- | --- | --- | --- | --- | --- |
| **MONITORING** | | | | | |
| Better monitoring in general | X | X | X |  | X |
| Continuous / constant monitoring | X | X | X |  |  |
| Opportunity of long-term monitoring | X |  |  |  |  |
| (Automatic) alerts on limit exceedances / approaches | X | X | X | X | X |
| **TREATMENT** | | | | | |
| Special application opportunities in treatment |  | X |  |  |  |
| Better treatment options | X | X | X | X | X |
| Earlier / faster treatment | X | X | X |  | X |
| More targeted treatment |  |  | X |  |  |
| Better overview of treatment progress |  | X |  |  | X |
| Easier adjustments in the treatment process |  | X |  |  |  |
| **PREVENTION** | | | | | |
| Special application opportunities in prevention |  | X |  |  |  |
| Possibility of more preventive work | X | X | X | X | X |
| Increasing prevention |  | X | X |  | X |
| Strengthening / increasing the focus on health (rather than illness) |  |  | X |  |  |
| Early detection before the onset of symptoms or deterioration | X | X |  |  | X |
| Use of prevention to limit the extent of treatment |  | X |  |  |  |
| **RESEARCH / SCIENTIFIC UNDERSTANDING** | | | | | |
| Great potential for research use | X |  | X |  |  |
| Advancing medical research | X | X | X | X |  |
| Defeating diseases | X |  |  |  |  |
| More empirical data |  |  |  | X |  |
| Improving the quality of research | X |  | X | X |  |
| Medical progress through individualisation and linking correlations | X | X |  | X |  |
| **DIAGNOSTICS** | | | | | |
| Special application opportunities in diagnostics | X | X |  |  | X |
| Increasing the diagnostic quality in general | X | X | X | X | X |
| Supplementing the anamnesis with a third method | X | X |  |  |  |
| Facilitating diagnoses | X | X | X | X | X |
| Faster diagnoses | X |  | X |  | X |
| Enabling diagnoses despite hidden symptoms | X | X |  |  |  |
| Early diagnoses | X | X | X |  | X |
| Access to patient data outside the patient’s room |  | X |  |  |  |
| Implementing individual parameters | X |  |  |  |  |
| Possibility to create a health profile | X |  |  |  | X |
| Possibility of predicting future health status | X |  |  |  |  |
| Secondary use of collected data | X |  |  | X |  |
