# Supplementary material for: Exploring Attitudes Toward AI-Based Contactless Sensors in Health Among Five Stakeholder Groups: Qualitative Study
Source: J Med Internet Res. 2026 Apr 24;28:e75783. doi: 10.2196/75783 (PMC13108836; doi:10.2196/75783)
Supplement: Multimedia Appendix 8 [file jmir-v28-e75783-s008.docx]

| **MEDICAL CHALLENGES** | Patients | Healthcare Professionals | Researcher | Political Stakeholder | General  Public |
| --- | --- | --- | --- | --- | --- |
| **MONITORING** | | | | | |
| Possibility of incidents despite regular monitoring |  | X |  | X |  |
| Use of other measurement methods in some cases medically more useful | X |  |  |  |  |
| Occurrence of medical harm due to radiation exposure |  |  | X |  |  |
| Causing anxiety in patients (by prescribing or measuring with sensors) |  |  |  | X | X |
| **TREATMENT** | | | | | |
| Incorrect treatments due to data / analysis errors or false alarms | X | X |  | X | X |
| Possibility of intervening too late (e.g. in the case of an attack or a fit) | X |  |  |  | X |
| Unequal treatment of patients on the basis of their choice of measurement method |  |  | X |  |  |
| Impeding the healing process of patients with loss of interpersonal relationships | X |  |  |  |  |
| **DIAGNOSTICS** | | | | | |
| Misdiagnosis due to incorrect measurements / incorrect learning data |  |  |  | X |  |
| Misdiagnosis due to (automated) misinterpretation of symptoms | X | X |  |  | X |
| Risk of premature diagnosis | X |  |  |  |  |
| Risk of false positive diagnosis |  |  | X |  |  |
| Risk of limiting health parameters to measured data only |  | X |  |  |  |
| Risk of unreliable data leading to false prognosis |  | X | X |  |  |
| Unintended / unwanted diagnosis of further diseases | X | X | X |  | X |
| Failure to recognise other symptoms due to complete reliance on sensor readings | X |  |  | X |  |
| Doctors' overconfidence in technology and lack of confidence in their skills |  |  | X |  |  |
| Patient uncertainty due to data flooding |  |  |  |  | X |
| Difficulty in defining necessary standard health parameters | X |  | X |  |  |
| Restriction of the doctor's medical activity |  | X |  | X |  |
| Questioning the diagnosis of mental disorders based on quantitative data | X | X | X |  | X |
| Questionable value of diagnosing patients at certain stages of life (e.g. dying stage) |  | X |  |  | X |
