# Supplementary material for: Exploring Attitudes Toward AI-Based Contactless Sensors in Health Among Five Stakeholder Groups: Qualitative Study
Source: J Med Internet Res. 2026 Apr 24;28:e75783. doi: 10.2196/75783 (PMC13108836; doi:10.2196/75783)
Supplement: Multimedia Appendix 9 [file jmir-v28-e75783-s009.docx]

| **TECHNICAL OPPORTUNITIES** | | Patients | Healthcare Professionals | Researcher | Political Stakeholder | General  Public |
| --- | --- | --- | --- | --- | --- | --- |
| **IMPLEMENTATION** | | | | | | |
| Simple installation | |  | X | X | X | X |
| Reduced system maintenance required once accuracy is proven | |  | X |  |  |  |
| Small size of the sensor | | X | X |  | X |  |
| **USE** | | | | | | |
| **Patient-Related** | | | | | | |
|  | Unconscious measurement |  | X |  |  | X |
|  | Avoidance of radiation exposure | X |  |  |  |  |
|  | Individualization |  |  | X |  |  |
| **Self-Application** | | | | | | |
|  | Self-measurement as a particular opportunity |  | X |  |  |  |
|  | Passivity of the patient |  | X | X | X | X |
|  | Flexibility of the measuring locations (e.g. in familiar surroundings) | X | X | X | X |  |
|  | Easy integration into everyday life |  | X | X | X | X |
| **Measurement** | | | | | | |
|  | Continuous measurement |  | X | X | X |  |
|  | Long-term measurement | X | X | X |  |  |
|  | Automatic measurement |  | X |  |  |  |
|  | Distance measurement | X | X | X |  | X |
|  | Measurement speed |  | X | X |  | X |
|  | Efficiency of measurement | X |  |  |  | X |
|  | Simplicity of measurement |  | X | X | X | X |
| **Measuring** | | | | | | |
|  | No wiring of patients required | X | X | X | X |  |
|  | More objective measurement through standardisation |  | X | X |  |  |
|  | Improved measurement accuracy | X |  |  | X |  |
|  | Improved measurement reliability | X |  | X |  |  |
|  | Avoidance of measurement deviations |  | X | X | X | X |
|  | More detailed / accurate documentation |  |  |  |  | X |
| **DATA** | | | | | | |
| **Collected Data** | | | | | | |
|  | Generation of large amounts of data | X | X | X | X |  |
|  | Generation of diverse data sets |  |  | X |  |  |
|  | Improved data quality (more meaningful / objective / reliable / realistic data) |  | X | X |  |  |
|  | Long-term and easy storage of numerical data |  |  | X |  |  |
|  | Anonymisation of data |  | X |  |  |  |
|  | Ability to monitor without data storage |  |  | X |  | X |
|  | Improved ability to exchange and communicate data | X |  |  |  |  |
| **Analysis of Data** | | | | | | |
|  | Improved analysis of data (through continuous / automatic analysis) |  | X | X |  |  |
|  | Improved analysis possibilities through AI | X |  |  |  |  |
|  | Online access to the device as an advantage for data analysis | X |  |  |  |  |
| **ADVANCES IN MEDICAL / TECHNICAL PROGRESS** | | | | | | |
| Measurement of previously unmeasurable movement patterns / diseases | | X | X | X | X |  |
| Better detection of both small / large movements as well as movement patterns | | X | X | X |  |  |
| Added value for already measurable parameters | | X | X |  |  |  |
| Recording of comparative skin images / screenings as an opportunity | | X |  |  |  |  |
| Possible applications mainly for diseases that can be measured quantitatively | | X |  |  |  |  |
| Additional toll / device for the collection and analysis of data | |  |  | X |  |  |
| More advanced and innovative measurement method | | X |  | X |  |  |
| Supporting tool / device in health | |  |  |  |  | X |
| Opportunity to become the gold standard | |  |  |  |  | X |
