# Supplementary material for: Exploring Attitudes Toward AI-Based Contactless Sensors in Health Among Five Stakeholder Groups: Qualitative Study
Source: J Med Internet Res. 2026 Apr 24;28:e75783. doi: 10.2196/75783 (PMC13108836; doi:10.2196/75783)
Supplement: Multimedia Appendix 10 [file jmir-v28-e75783-s010.docx]

| **TECHNICAL CHALLENGES** | | Patients | Healthcare Professionals | Researcher | Political Stakeholder | General  Public |
| --- | --- | --- | --- | --- | --- | --- |
| **IMPLEMENTATION** | | | | | | |
| Possible incompatibility with other systems | |  | X |  | X |  |
| Installation required | |  | X |  |  |  |
| Complex adjustment of the devices to individual reference values | | X | X |  |  |  |
| Difficulty int he calibration of a contactless sensor | |  |  |  |  | X |
| Intensive training of medical staff required | |  |  |  |  | X |
| Lack of competence in application | |  |  |  | X |  |
| **USE** | | | | | | |
| **Patient-Related** | | | | | | |
|  | (Low) Radiation exposure |  | X | X |  |  |
|  | Uncertainty of behaviour during measurement |  |  |  | X |  |
|  | Unfamiliar (and perhaps frightening) form of monitoring for patients |  | X |  |  | X |
|  | Lack of insight into and knowledge about measurement time and data |  | X |  | X |  |
|  | Difficult to control by patients |  | X |  |  | X |
|  | No interruption of data collection possible (e.g. in intimate moments) |  |  |  | X |  |
| **Self-Application** | | | | | | |
|  | Difficulties in use | X |  |  |  |  |
|  | Interruption of continuous measurement for various reasons |  |  |  | X |  |
|  | Portability of sensors as a challenge (e.g. risk of loss) | X |  |  | X |  |
|  | Data easily falsified |  |  |  |  | X |
| **Reliable functionality** | | | | | | |
|  | System failures | X | X | X |  | X |
|  | False alarms |  |  | X |  |  |
|  | Difficult to ensure failsafe data collection | X |  |  |  | X |
|  | Closed-loop control of the system |  | X |  | X |  |
| **Measurement** | | | | | | |
|  | Reliability of measured data questioned |  |  | X |  | X |
|  | Accuracy of measured data questioned |  | X | X | X | X |
|  | Collection of unnecessary data | X |  |  | X |  |
|  | Technical limitations of contactless measurement |  |  | X |  |  |
| **Measuring** | | | | | | |
|  | Application errors |  |  | X | X |  |
|  | Bycatch |  |  | X |  | X |
|  | Incorrect measurement results due to external influences | X | X |  | X | X |
|  | Distorted measurement results due to the conscious adaptation of patient behaviour |  |  | X |  |  |
|  | Increased data distortion due to data visualisation |  |  | X |  |  |
|  | Intentional falsification of data by patients unlikely |  |  | X |  |  |
| **DATA** | | | | | | |
| **Collected Data** | | | | | | |
|  | Visual representation of collected data for patients |  |  | X |  |  |
|  | Limited meaningfulness / comparability of data (due to parameter individuality) | X |  |  |  |  |
|  | Falsification of data (due to various sources of measurement error) |  |  |  |  | X |
|  | Biased data sets |  |  |  | X |  |
|  | Data storing and data management |  |  |  |  | X |
|  | High potential for data misuse |  |  |  | X |  |
|  | Risk of sensitive data being hacked | X | X |  |  |  |
|  | Risk of data interception during data transfer | X |  |  |  |  |
| **Analysis of Data** | | | | | | |
|  | Difficult to analyse due to large amount of data from continuous measurement |  | X | X | X | X |
|  | Errors in data analysis due to errors in the system or non-diverse data sets |  |  |  | X |  |
|  | Data interpretation by AI must first be learnt |  | X |  | X |  |
| **DEVELOPMENT** | | | | | | |
| Technical development of sensors not sufficiently mature | |  | X | X |  | X |
| Lengthily development time due to developers lacking application experience | |  | X |  |  |  |
| Technical feasibility questioned | |  |  | X |  |  |
| Lack of long-term research funding as an obstacle to development | |  | X |  |  |  |
