# Supplementary material for: Exploring Attitudes Toward AI-Based Contactless Sensors in Health Among Five Stakeholder Groups: Qualitative Study
Source: J Med Internet Res. 2026 Apr 24;28:e75783. doi: 10.2196/75783 (PMC13108836; doi:10.2196/75783)
Supplement: Multimedia Appendix 11 [file jmir-v28-e75783-s011.docx]

| **ECONOMIC OPPORTUNITIES** | Patients | Healthcare Professionals | Researcher | Political Stakeholder | General  Public |
| --- | --- | --- | --- | --- | --- |
| **WITH REGARD TO HEALTH SYSTEM** | | | | | |
| Development and modernisation |  | X | X | X |  |
| Increasing efficiency |  | X | X |  | X |
| Cost savings | X |  | X | X |  |
| Improving service provision in healthcare | X |  | X | X | X |
| **WITH REGARD TO MEDICAL STAFF** | | | | | |
| Time savings for medical staff | X | X | X |  | X |
| Savings in personnel resources | X |  | X |  |  |
| Possible solution to the shortage of medical staff | X |  |  |  | X |
| **WITH REGARD TO PATIENTS** | | | | | |
| Time savings for patients | X | X | X |  | X |
| More time for patient concerns during doctor's visits |  | X | X |  |  |
| **WITH REGARD TO THE PRODUCTION OF SENSORS** | | | | | |
| Affordability | X |  | X |  | X |
| Competition from different providers |  |  | X |  |  |
| Faster ways to produce |  |  | X |  |  |
| Faster deployment of devices possible |  |  | X |  |  |
