# Supplementary material for: Exploring Attitudes Toward AI-Based Contactless Sensors in Health Among Five Stakeholder Groups: Qualitative Study
Source: J Med Internet Res. 2026 Apr 24;28:e75783. doi: 10.2196/75783 (PMC13108836; doi:10.2196/75783)
Supplement: Multimedia Appendix 12 [file jmir-v28-e75783-s012.docx]

| **ECONOMIC CHALLENGES** | Patients | Healthcare Professionals | Researcher | Political Stakeholder | General  Public |
| --- | --- | --- | --- | --- | --- |
| **WITH REGARD TO HEALTH SYSTEM** | | | | | |
| Increased performance pressure for hospitals / healthcare systems |  |  | X |  |  |
| Lack of basic structures in hospitals |  |  |  | X |  |
| Development of sensor farms in hospitals for mass monitoring |  |  | X |  |  |
| Use of sensors for economic reasons without medical indication | X | X | X |  |  |
| **WITH REGARD TO MEDICAL STAFF** | | | | | |
| Tying up medical staff resources |  |  |  | X | X |
| Increasing staff overload (instead of staff relief) |  | X |  | X | X |
| Staff reduction through the use of sensors to maximise profits |  | X | X |  | X |
| **WITH REGARD TO PATIENTS** | | | | | |
| Reduced time spent with patients |  |  | X |  | X |
| Pressure on patients to take responsibility for reducing healthcare costs |  |  |  |  | X |
| **WITH REGARD TO THE PRODUCTION OF SENSORS** | | | | | |
| Lack of long-term research funding as a barrier to development |  | X |  |  |  |
| Quality differences between sensors from different suppliers |  |  | X |  |  |
| Rejection of over-the-counter sale of sensors |  |  |  |  | X |
| **WITH REGARD TO HEALTH INSURANCES** | | | | | |
| (Fear of) individualised health insurance premium contributions |  |  | X | X |  |
| Risk of abandoning solidarity-based health insurance systems |  |  |  |  | X |
| Patient transparency through massive data collection and storage |  | X |  |  | X |
| **NON-HEALTH-RELATED DATA USE** | | | | | |
| Use of commercial data as a risk in itself | X |  |  |  | X |
| Refusal to use data outside the health sector | X |  |  | X | X |
| Lack of transparency on how companies use and process data |  | X |  | X |  |
| Large-scale data ownership by tech giants |  |  | X |  |  |
