# Supplementary material for: Exploring Attitudes Toward AI-Based Contactless Sensors in Health Among Five Stakeholder Groups: Qualitative Study
Source: J Med Internet Res. 2026 Apr 24;28:e75783. doi: 10.2196/75783 (PMC13108836; doi:10.2196/75783)
Supplement: Multimedia Appendix 13 [file jmir-v28-e75783-s013.docx]

| **SOCIO-POLITICAL OPPORTUNITIES** | Patients | Healthcare Professionals | Researcher | Political Stakeholder | General  Public |
| --- | --- | --- | --- | --- | --- |
| **SOCIAL OPPORTUNITIES** | | | | | |
| Positive social benefit through utilisation | X |  | X |  | X |
| Accessibility for everyone |  |  | X |  |  |
| Improving communication |  | X | X |  | X |
| Relief for relatives |  |  | X |  | X |
| **POLITICAL OPPORTUNITIES** | | | | | |
| Acceptance of use |  | X | X | X |  |
| Potential for managing demographic change |  |  |  | X |  |
| Removing barriers | X | X |  |  |  |
| Civil protection |  | X | X |  |  |
| An opportunity in itself: balancing risk and opportunity |  |  | X |  |  |
