# Supplementary material for: Exploring Attitudes Toward AI-Based Contactless Sensors in Health Among Five Stakeholder Groups: Qualitative Study
Source: J Med Internet Res. 2026 Apr 24;28:e75783. doi: 10.2196/75783 (PMC13108836; doi:10.2196/75783)
Supplement: Multimedia Appendix 14 [file jmir-v28-e75783-s014.docx]

| SOCIAL CHALLENGES | Patients | Healthcare Professionals | Researcher | Political Stakeholder | General  Public |
| --- | --- | --- | --- | --- | --- |
| **INDIVIDUAL RISKS** | | | | | |
| Social consequences of increasing contactlessness |  | X |  |  |  |
| Loneliness |  | X |  |  |  |
| Risk of seeing oneself as superfluous | X |  |  |  |  |
| Overconcentrating on own medical data |  |  |  |  | X |
| Decreased awareness of one's own body | X |  |  |  |  |
| Increasing pressure to meet the expected data parameters | X |  |  |  | X |
| Pressure for self-optimisation |  | X |  |  | X |
| Increases in patient anxiety / stress |  | X | X |  | X |
| Risk of accelerating life | X |  |  |  |  |
| **FEARS** | | | | | |
| Fear of surveillance and anxiety caused by surveillance measures | X | X |  |  | X |
| Fear of losing touch |  | X | X |  |  |
| Fear due to invisibility of measurement |  | X |  | X |  |
| Fear of radiation | X | X | X |  |  |
| Fear of electric shocks | X |  |  |  |  |
| Fear of people being reduced to numbers and facts |  |  | X |  |  |
| Fear of having fundamental rights violated | X |  |  | X |  |
| Fear of (social) selection | X |  |  |  |  |
| Fear of job cuts |  | X |  |  |  |
| **INTERACTION** | | | | | |
| Loss of interpersonal and psychosocial interaction | X | X | X |  | X |
| Loss of the human connection | X |  | X |  |  |
| Loss of empathy |  |  | X |  |  |
| Need for interpersonal interaction (for certain diseases / medical specialities) | X |  | X |  |  |
| Medical staff focused on machines rather than patients |  | X |  |  | X |
| Diffused responsibility of medical staff |  |  |  | X |  |
| Social pressure on patients when medical staff insist on a measurement |  | X |  |  |  |
| Reliability of measurement or doctor visit associated with touch |  |  | X |  |  |
| Influencing the development of a society |  |  | X |  |  |
| Challenging social norms and concepts of society | X |  | X |  |  |
| Restriction of social participation |  |  | X |  | X |
