# Supplementary material for: Exploring Attitudes Toward AI-Based Contactless Sensors in Health Among Five Stakeholder Groups: Qualitative Study
Source: J Med Internet Res. 2026 Apr 24;28:e75783. doi: 10.2196/75783 (PMC13108836; doi:10.2196/75783)
Supplement: Multimedia Appendix 15 [file jmir-v28-e75783-s015.docx]

| **POLITICAL CHALLENGES** | Patients | Healthcare Professionals | Researcher | Political Stakeholder | General  Public |
| --- | --- | --- | --- | --- | --- |
| **ACCEPTANCE** | | | | | |
| Expected low acceptance and difficulties in accepting |  | X | X | X |  |
| Society's discomfort with radiation |  | X | X |  |  |
| Influence of different educational backgrounds on acceptance | X |  |  |  |  |
| Various reasons for refusal (lack of knowledge, personal preferences, etc.) | X | X |  |  |  |
| Lack of education and public information |  |  | X | X | X |
| **PREIMPLEMENTATION CONSIDERATIONS** | | | | | |
| Limited benefits |  |  | X |  | X |
| Challenging assessment of the advantages and disadvantages of sensor use |  | X |  | X | X |
| Power of the insurance companies to decide on the use of the sensors |  |  |  |  | X |
| **IMPLEMENTATION** | | | | | |
| Long implementation time of new technologies in healthcare systems |  |  | X | X |  |
| Nationwide availability of sensors |  | X |  |  |  |
| Lack of cultural change from a reactive to a preventive healthcare system |  |  |  | X |  |
| Patient-related and process-related difficulties |  | X |  | X |  |
| Uncertainty about unforeseen consequences of use |  |  |  | X | X |
| Need to clarify sensor distribution, prescription and billing responsibilities |  |  |  | X |  |
