# Supplementary material for: Exploring Attitudes Toward AI-Based Contactless Sensors in Health Among Five Stakeholder Groups: Qualitative Study
Source: J Med Internet Res. 2026 Apr 24;28:e75783. doi: 10.2196/75783 (PMC13108836; doi:10.2196/75783)
Supplement: Multimedia Appendix 16 [file jmir-v28-e75783-s016.docx]

| **REGULATORY CHALLENGES** | Patients | Healthcare Professionals | Researcher | Political Stakeholder | General  Public |
| --- | --- | --- | --- | --- | --- |
| **ABUSE OF SENSORS** | | | | | |
| Fine line between use and potential abuse | X |  |  | X |  |
| High risk of abuse due to invisibility of the measurement | X | X | X |  |  |
| Dual-use risk (high potential for misuse outside the health sector) | X | X | X | X | X |
| Increased surveillance possibilities (by states / employers / individuals) | X | X | X |  | X |
| Misuse by states / employers to control citizens / employees |  |  | X | X | X |
| Risk of excessive control |  |  |  | X |  |
| **CONSENT** | | | | | |
| Being measured unknowingly (e.g. bycatch) |  | X | X |  | X |
| Being measured without consent | X | X |  | X | X |
| Difficulty in obtaining informed consent from certain patient groups |  | X | X | X | X |
| Patient involvement |  | X |  |  |  |
| Enabling choice in consent as a major effort |  | X | X |  | X |
| Dealing with rejection of the technology |  | X |  |  | X |
| Patients' sense of uncontrollability of the measurements |  |  | X |  | X |
| Lack of understanding of the consequences of one's own consent |  |  | X |  |  |
| **ABUSE OF DATA** | | | | | |
| Large scope for data abuse in general (by companies / employers / individuals / etc.) |  | X | X |  | X |
| Hacking / phishing |  |  |  |  | X |
| Interception of data |  |  | X |  |  |
| Unauthorised disclosure of data |  | X | X |  | X |
| Unauthorised secondary use of data already collected |  |  |  | X |  |
| Attribution of even anonymised data to individuals (e.g. based on gait) |  |  |  | X |  |
| **REGULATION** | | | | | |
| Regulation as a general challenge | X |  |  | X | X |
| Challenge of regulating and approving digital medical devices |  |  |  | X |  |
| Legal admissibility of monitoring / recording moving patterns questioned |  | X |  | X |  |
| Limited effectiveness of laws and ethical standards to prevent abuse |  | X |  |  | X |
| Liability / accountability / responsibility in the event of damage / harm |  |  |  | X | X |
| Ambivalence between intended awareness /law) & non-awareness (measurement) |  |  | X |  |  |
| **GENERAL DATA-RELATED CHALLENGES** | | | | | |
| Data control |  | X | X |  | X |
| Data protection / data security | X | X | X |  |  |
| Data ownership | X |  |  |  | X |
